# Chemical Sensor Systems and Associated Algorithms for Fire Detection: A Review

**DOI:** 10.3390/s18020553

**Published:** 2018-02-11

**Authors:** Jordi Fonollosa, Ana Solórzano, Santiago Marco

**Affiliations:** 1 Department of Electronic and Biomedical Engineering, Universitat de Barcelona, 08028 Barcelona, Spain; asolorzano@ibecbarcelona.eu (A.S.); smarco@ibecbarcelona.eu (S.M.); 2 Signal and Information Processing for Sensing Systems, Institute for Bioengineering of Catalonia (IBEC), The Barcelona Institute of Science and Technology, 08028 Barcelona, Spain; 3 Department of ESAII, Center for Biomedical Engineering Research, Universitat Politècnica de Catalunya, 08028 Barcelona, Spain

**Keywords:** fire detection, gas sensor, pattern recognition, sensor fusion, machine learning, toxicants, carbon monoxide, hydrogen cyanide, standard test fires, transducers, smoke

## Abstract

Indoor fire detection using gas chemical sensing has been a subject of investigation since the early nineties. This approach leverages the fact that, for certain types of fire, chemical volatiles appear before smoke particles do. Hence, systems based on chemical sensing can provide faster fire alarm responses than conventional smoke-based fire detectors. Moreover, since it is known that most casualties in fires are produced from toxic emissions rather than actual burns, gas-based fire detection could provide an additional level of safety to building occupants. In this line, since the 2000s, electrochemical cells for carbon monoxide sensing have been incorporated into fire detectors. Even systems relying exclusively on gas sensors have been explored as fire detectors. However, gas sensors respond to a large variety of volatiles beyond combustion products. As a result, chemical-based fire detectors require multivariate data processing techniques to ensure high sensitivity to fires and false alarm immunity. In this paper, we the survey toxic emissions produced in fires and defined standards for fire detection systems. We also review the state of the art of chemical sensor systems for fire detection and the associated signal and data processing algorithms. We also examine the experimental protocols used for the validation of the different approaches, as the complexity of the test measurements also impacts on reported sensitivity and specificity measures. All in all, further research and extensive test under different fire and nuisance scenarios are still required before gas-based fire detectors penetrate largely into the market. Nevertheless, the use of dynamic features and multivariate models that exploit sensor correlations seems imperative.

## 1. Introduction

Nowadays, the most popular and widespread fire alarm systems are based on the detection of smoke. Two techniques for smoke detection emerge for fire detection: photoelectric detectors (light scattering) and ionization detectors. However, the search for alternative detection techniques to improve occupant’s safety and reduce the number of false alarms is an active field of research.

Fire detection using gas sensing has been recognized as a promising approach since the 1990s. First, fire detection based on chemical sensing could provide faster alarm signals when gases are released before smoke particles. Second, chemical-based fire detection could offer additional safety to building occupants as it is known that most casualties in fires are produced from toxic emissions rather than actual burns [1]. However, the current use of fire detection systems based on gas sensors has been limited to niche scenarios, such as fire detection in coal mines [2] or coal power plants [3].

Light scattering and ionization detectors are not sensitive to toxic emissions, so they may not offer enough protection in the case of smoldering fires. In terms of time response, it can take a long time from when a fire starts until conventional smoke-detectors trigger the alarm, when dangerous toxic gas concentrations may have already reached levels that threaten people’s lives [4].

The importance of toxic emissions in fires has been recognized as a primary hazard for building’s occupants since the 1970s, when surveys about fire deaths and non-fatal fire injuries were carried out in the UK. These surveys showed that a substantial proportion of casualties was due to fire emissions and not to actual burns. Additionally, the same studies demonstrated that the fraction of deaths due to toxic emissions was growing over time (a fourfold increase from the 50s to the 70s). This increasing trend continued during the 80s and 90s, although the overall number of fires remained approximately constant in that period. For example, during the 90s, only in the UK, the total number of injuries attributed to toxic fire emissions was about 6000 per year, and the total number of deaths was about 14/million inhabitants/year. The increase of injuries caused by toxic emissions in fires has been attributed to the increasing popularity of polymers in building materials, with the underlying idea that new building materials produce more toxic effluents than conventional materials. Other interpretations claim that the released toxic gases are the same ones for new and conventional materials, but volatiles are released at a much higher pace from new materials [5]. Hence, high concentration levels of toxicants can be released in fire situations nowadays, threating occupants’ health even before smoke is detected by conventional fire detectors.

Additionally, smoke detectors are unable to discriminate between smoke particles from fires and particles from other events, leading to high rate of false positives. False alarms are always a concern for fire detection due to high associated costs and frequency. Only in the UK, for example, the Fire and Rescue Service Authorities claim that the associated cost of false alarms rises to 1 billion pounds per year [6]. The same source claims that in the period 2011–2012, 53% of the alarms were false positives. Moreover, even worse ratios of false alarms have been reported in studies performed in the 90s in Europe and the US. In some reports, the fraction of real alarms was as low as 11% [7]. Indeed, there are many daily activities that may lead to false alarms (nuisances), being burning toasts and cooking fumes in general, dust from building works, water steam from the shower, etc., examples of the most prevalent ones.

In fact, it has been long found that it is difficult to discriminate nuisances from early fire by processing data from a single sensor [8]. In order to improve the reliability of fire detectors, multisensor systems including heat, CO electrochemical cells, and smoke detectors have been explored over the years [9]. As a result, more sophisticated fire detectors that use several types of sensors, or sensors located at different points have been proposed. Such multisensor systems can also benefit from algorithms built for single-sensor systems, as decision rules based on logic rules can be combined with the different sensors, but tailored algorithmic solutions to build calibration models for multisensor systems are more common than the extension of single sensor solutions to multiple sensor systems.

The reliability of fire predictions was successfully improved when heat and CO sensing was added to smoke detectors and they were combined with dedicated calibration models. Standardized tests for such kind of multisensor systems are available. However, different approaches based on non-specific gas sensors and other sensing devices have been proposed to reduce the costs and consider other combustion products beyond CO. These non-standardized systems have been subject of investigation by the community as they can detect more toxicants and combustion products and can provide faster detection, although they suffer from low specificity.

To build robust and reliable fire alarm systems, multisensor systems need to be exposed to many types of fires and nuisances. The quality of the classification model depends critically on the number and conditions of the considered fires and nuisances. However, the benchmark of the different systems is difficult due to the disparity of experimental setups and difficulties and cost of data generation. In this work, we will focus on the challenges and opportunities offered by fire detectors that include chemical sensors. In particular, we will focus on their ability to act as reliable fire detectors and their potential to detect toxic emissions that may appear in the early phases of fire development.

The organization of the paper is as follows: In Section 2, we review conventional detectors based on smoke detection. In Section 3, we cover the gas emissions in fires and their toxicity. We briefly review models to estimate the toxic potency of multi-component gases from fires. In Section 4, we cover how the different fire scenarios and burning materials determine gas emissions, and we show some examples focusing mostly on smoldering fires. In Section 5, we briefly review current international standards for fire sensitivity testing. Then in Section 6, we cover the basic technologies available for chemical sensing in regard to low-cost fire detection. In Section 7 we cover the state of the art concerning algorithms used to detect fires and reject nuisances. Finally, Section 8 summarizes our review.

## 2. Fire Detectors Based on Smoke Detection

Nowadays, the most popular and widespread fire alarm systems are based on the detection of smoke. Smoke is defined as “*the airborne solid and liquid particulates and gases evolved when a material undergoes pyrolysis or combustion*” [10]. However, in this context, smoke detectors refer exclusively to the detection of fire particulates, excluding gas detection. Two techniques for smoke detection emerge for fire detection: Photoelectric detectors (light scattering) and ionization detectors. Briefly, ionization smoke alarms use a radioactive source, usually Americium-241, that emits alpha particles to ionize air molecules. The generated ions close the path of an electric circuit. If smoke is present, the generated ions interact with smoke particles, reducing thereby the intensity that flows through the circuit. The need for a radioactive emitter to break the molecules into ions has decreased the popularity of ionization detectors. On the other hand, photoelectric detectors include a light emitter and a photo-detector. If there is smoke in the chamber, smoke particles produce light scattering. Scattering or obscuration of light is measured with the detector. Typically, independently of the detection principle, the alarm signal is triggered when the signals reach some defined threshold.

The sensitivity, response time and reliability of the fire alarm usually depend on the sensing principle. In order to establish formal benchmarks between sensing principles, photoelectric and ionization fire alarms were compared extensively in controlled conditions [11]. Such studies suggest that usually, ionization alarms respond faster than photoelectric alarms to open flame fires. In contrast, photoelectric alarms tend to show faster response and higher sensitivity than ionization detectors in smoldering fires. For example, Underwriters Laboratories Inc. compared photoelectric and ionization detectors under different fire types inspired by the UL 217 standard (see Section 5) and other fires [12]. Flaming and smoldering fires produced combustion particles of different diameter, which conditioned the response of the different detectors. Smoldering fires produced larger particles, which were captured faster by photoelectric detectors. On the other hand, smaller particles, which are found in flaming fires, were detected faster by ionization detectors. Moreover, the results indicated that, given the same consumed mass, smoldering fires resulted in more smoke particles than flame fires. They also found that ionization alarms could not detect some smoldering fires that photoelectric alarms detected. This became more relevant for smaller burning quantities that generated less smoke than the 10% obscuration/ft specified in the UL 217 standard.

Briefly, smoke detectors can be considered as particle detectors that are sensitive to a specific distribution of particle sizes. Usually, fire alarm is triggered when the sensor signal reaches an established threshold. As a result, these systems struggle in discriminating particles resulting from fires and non-combustion particles when the particles have similar size or refractive indices. For example, smoke detectors also show sensitivity to water vapor and dust [13]. Moreover, they cannot distinguish combustion products from a fire threat condition from combustion products produced under controlled conditions, such as cigarette smoke or some cooking activities [14].

In summary, both photoelectric and ionization fire alarm systems show cross-sensitivities that yield false alarms. The false alarm ratio sometimes becomes too high for the resident, who is then tempted to disable or ignore fire alarm signals.

In order to improve the specificity of the fire alarm, other sensors can be added to smoke detectors. For example, common nuisance scenarios such as cooking aerosols, water steam (from cooking or showers) and dust sources increase light obscuration but do not result in CO concentration increases. Hence, CO detection can be used to improve false alarm immunity and reject false alarms induced in scenarios that do not generate CO [15].

Unlike smoke-based fire alarms, systems based on single measurements from one gas sensor would not be suitable for fire detection, as the number of false alarms would be unacceptably high. For example, fire detection system based on single CO measurements would overlook flame fires and would be sensitive to exhaust gases from gas or oil furnaces. As a result, gas-based systems require multiple sensor or multi-criteria approaches, and, thereby, more complex data processing algorithms.

## 3. Gas Emission in Fires

A wide variety of materials are found nowadays inside occupied buildings. The burning of these materials results in the release of different combustion products, namely aerosols and gases. Additionally, products not actually burning may reach temperatures high enough to suffer from thermal decomposition and pyrolysis, producing thereby additional emission of gases and volatiles [10]. All these products constitute health hazards for building occupants and emergency personnel.

Health hazards may be divided into several categories:

1. Irritants: Fire gases and particles producing irritation of the respiratory tract, that can impair the ability to escape and, at higher concentrations, can lead to incapacitation and death.
2. Asphyxiants: Inhalation of these gases can produce the depression of the central nervous system leading to disorientation, loss of coordination, loss of conscience and finally death.
3. Thermal effects: Thermal burns on the skin and the respiratory tract, as well as hyperthermia.

When exposed to the above-mentioned hazards, the impact on the building occupant’s health depends on the previous health condition of the individual (age, morbidities, asthma, etc.) and on the nature of the exposure: exposure time, gas concentration, toxicity of the volatiles, etc. Moreover, the incapacity to find the escape path due to eye irritation and smoke obscuration produces longer exposures to these hazards. Fire survivors can also suffer from post-exposure and delayed health effects.

Emissions of gases and volatiles may occur during pyrolysis or during combustion. Pyrolysis is defined as “*a process of simultaneous phase and chemical species change caused by heat*”, while combustion is “*a chemical process of oxidation that occurs at a rate fast enough to produce temperature rise and usually light, either as glow or flame*” [10].

Since the 1980s, the use of polymeric materials in commercial products has increased dramatically. This results in more volatile emissions during fires: when heated, polymeric materials may show phase change (melting in thermoplastics) followed by thermal decomposition. This leads to the emission of low weight volatile compounds, prior to actual combustion happens and before visible smoke appears.

Gas emissions are also particularly relevant during smoldering fires. This is a form of combustion that mostly occurs in porous or grained but densely packed materials. Air diffuses through the pores and produces combustion in the inner side of the material. The combustion products in smoldering fires are typically different from the ones generated in open flame fires.

In smoldering fires, the temperature is low (around 400 °C) and fire materials decompose due to a combination of pyrolysis and oxidation. In this type of fires, the CO/CO_2_ ratio is close to 1, and CO may be the major toxicant to consider. Fire evolution is slow, temperatures are also low, and the smoke density is not dense. Under these conditions, the occupants may die from asphyxia, particularly if they are asleep. In fact, it is known that smoldering fires that have been running for 30 min or more before being detected produce more casualties than fires that produce rapid flame fires [5].

### 3.1. Main Toxicants from Fire Emissions

A review of the literature will easily show that fires may emit hundreds if not thousands of gases and volatile compounds, however few of these are particularly relevant due to either their volume or their toxicity. Current understanding of fire emissions concludes that carbon monoxide is still today the main toxic component in fires. However, the presence or addition of other toxics may lead to much faster death than when only the effect of CO is considered. As already mentioned, the presence of synthetic polymers in building materials and building contents (for instance, electronics, cables, electrical appliances, etc.) is more and more determinant for toxic emissions since many of these materials contain nitrogen or halogen compounds, leading to the presence of hydrogen cyanide (HCN) and inorganic acids. Stec remarked that CO is not the only toxic gas released in fires. She studied other toxicants, in particular, the significance of HCN from PVC fires. Her results confirmed the danger of HCN in under-ventilated conditions [16]. Finally, oxygen depletion to 10% or lower usually increases the effects of the toxicants.

#### 3.1.1. Carbon Dioxide

CO_2_ is probably the most important combustion product. If there is enough ventilation, almost all carbon content is converted to CO_2_. The toxicity of CO_2_, individually is low, but as we will review in the next section, it can interact with other toxics exacerbating their effect.

#### 3.1.2. Carbon Monoxide

Carbon monoxide is an asphyxiant gas. CO emissions are particularly relevant in smoldering fires. For example, in many fires, CO is emitted and then it is oxidized to CO_2_. However, in the absence of sufficient ventilation, the second step is not efficient and larger concentrations of CO are found. In typical scenarios, lethal concentrations of CO may be reached close to the fire in less than 30 min. Moreover, after dilution, lethal concentrations may be reached in 1–2 h in the whole room.

The emission of CO is related to the air-fuel ratio (equivalence ratio) [17]:

(1) ϕ=m_fuelm_air(m_fuelm_air)stoich

where *m_fuel* is the mass of fuel, *m_air* is the mass of air, and *stoich* refers to the stoichiometric conditions. When a fire happens in stoichiometric conditions (*ϕ* = 1), there is exactly enough air to burn all the fuel. For *ϕ* < 1, fire conditions are considered *rich*, while for *ϕ* > 1 conditions are considered *lean*. Lean conditions provide higher production of CO.

#### 3.1.3. Hydrogen Cyanide

Hydrogen Cyanide is an asphyxiant gas. HCN originates from nitrogen-rich polymers such as wool, nylon, polyacrylonitrile, melamine, etc. The formation of this compound is not as well understood as the mechanism for CO formation, but in any case, its production is also enhanced in lean conditions. In the recent years, there has been an increasing concern on the relevance of this compound in mission intoxications of firefighters [18].

#### 3.1.4. Nitrogen Oxides

Fire effluents analyzed by FTIR have shown that nitrogen oxides appear mostly in the form of nitric oxide (NO). This gas is stable at the low concentrations and low temperatures typical of actual inhalation by humans in fire incidents. Nitric oxide also appears in tobacco smoke and in exhaust gases from motor vehicles. Alternatively, we may also find nitrogen dioxide (NO_2_). NO_2_ is highly soluble in water and it is an acid irritant with highly toxic effects. It has a higher toxic potency than NO [19].

#### 3.1.5. Sulphur Dioxide

Sulphur dioxide (SO_2_) is an irritant gas. It may appear in the combustion of some textiles like wood or viscose [20], but also rubber materials. Mathematical models of lethal toxicity of fire smoke consider sulphur dioxide a key component [21]. Sulphur dioxide has been detected in real overhaul operations in concentrations of around 2 ppm, with maximum values of 8.7 ppm.

#### 3.1.6. Halogen Acids

Halogen acids appear from the combustion of polymers containing halogen elements (fluorine, chlorine, bromine). Examples are polyvinylchloride (PVC), neoprene, polyvinyl fluoride, polytetrafluoroethylene, and brominated flame retardants. The most relevant ones are consequently hydrogen fluoride (HF), hydrogen chloride (HCl) and hydrogen bromide (HBr). These compounds appear mostly in the pyrolysis phase before actual combustion. Their concentration may be high since the efficiency of their production is very high. For instance, HCl is produced by PVC at temperatures between 225 to 275 °C [22].

#### 3.1.7. Organic Irritants

Incomplete combustion and pyrolysis of organic materials can produce a large variety of Volatile Organic Compounds (VOCs). The most toxic ones are considered to be formaldehyde, unsaturated aldehydes like acrolein and isocyanates [23]. Acrolein can be emitted, among other materials, from polyethylenes [24].

### 3.2. Toxicity of Released Gases in Fire

In this section we will review the main toxic mechanism of gases released in fires. As we have already mentioned, the toxic effects of fire gas emissions can be grouped in asphyxiants and respiratory irritants. Since, in the particular scenario of fires, the concentration of toxic gases is relatively high for a short period of time, the typical threshold limit values (TLV) used in occupational hygiene are not normally used.

#### 3.2.1. Asphyxiant Gases

**Carbon monoxide** is the most important and studied toxic emission from fires. The toxic effects that produce incapacitation, first, and ultimately death is related to the combination of CO with hemoglobin to form carboxyhemoglobin (COHb). Hence, COHb is a biomarker of smoke inhalation that can be used to investigate cause of death in fires. To determine if CO intoxication has been the main cause of death, COHb in blood is measured during forensic investigations. Usually it is considered that if COHb reaches 50% (normalized to the total hemoglobin content) death has been caused by CO inhalation during fire. An increase of COHb diminishes the capacity of blood to transport oxygen. Additionally, at elevated levels of COHb, there is a shift in the equilibrium reaction of HbO_2_ that hinders oxygen to be delivered to cells. Finally, when CO combines with myoglobin the transport of oxygen to muscle tissues (including cardiac) is reduced.

The Coburn-Forster-Kane (CFK) equation describes the dynamics of COHb formation from CO inhalation [25]. This model has been thoroughly validated and information on the population distribution of its parameters has been largely studied [26,27]. Additionally, it has been refined to include the decrease in HbO_2_ when COHb increases. This effect can be neglected at low CO concentrations but it becomes relevant at high CO and O_2_ depletion, as it happens in fire scenarios [28]. The CFK model is recommended to simulate the evolution of COHb and compute the time to incapacitation (30% COHb) and the time to lethal conditions (COHb > 50%). In this kind of simulation, a critical parameter in the CFK equation is the alveolar ventilation and the lung-diffusing capacity for CO, depending on the oxygen input flow. This input flow can change form 8 L/min at rest up to 100 L/min when escaping fast [29,30]. Under the academic hypothesis of a constant CO concentration, the Stewart-Peterson [31] equation provides the time required to reach a certain level of COHb:

(2) $$\mathrm{COHb}=B{\left[\mathrm{CO}\right]}^{1.036}\cdot \mathrm{RMV}\cdot t$$

where COHb is in %, CO in ppmv, respiratory minute volume (RMV) in L/min and t in min. With these units B = 3.32 × 10^−5^. The CO concentration that is accumulated in the blood in the form of COHb determines the effects on the subject. First symptoms in humans (headache) are reported when COHb reaches values of about 10%, after the subjects were exposed to 15,000 ppm of CO for 2 min, or 30,000 ppm for one minute. Time to incapacitation depends on the accumulated CO and also on the physical activity of the subject. For example, at 10,000 ppm of CO, probable time to incapacitation for humans was estimated at 10 min, 4 min, or 1 min for resting state, light work, or slow running, respectively. Additionally, it is interesting to remark that the half recovery time for adults at rest is 320 min [31,32].

**Carbon dioxide.** CO_2_ is not considered a toxic gas, but at high concentration levels, it increases the breath frequency and depth, leading to increased RMV. For instance, an atmosphere with 10% of CO_2_ concentration induces a 10-fold increased RMV on the exposed subject with respect to RMV in a not contaminated atmosphere. As a result, increased RMV produces faster intoxication by other gases and VOCs [33].

**Oxygen** depletion from 20.9% to 17% produces a degradation in motor coordination and, up to 10% of oxygen concentration, the exposed subject may still be conscious but will suffer incapacitation effects in terms of impaired judgment and fast fatigue conditions. From 10% to 7%, the person may lose consciousness. These conditions with very low oxygen concentration levels are only reached very close to flames, where heat is additionally the most important threat. Far from the fire flames, the most important effect of oxygen depletion is the combined effect with toxicants, for instance by augmenting the breath rate that leads to faster dynamics in the uptake of other toxics.

**Hydrogen Cyanide (HCN)** is lethal at doses much smaller than carbon monoxide and its toxic effects are very fast [34]. Like COHb, it can also be determined in blood to investigate its relevance in the event of death. In fact, HCN in blood is routinely found in forensic investigations of fire. Levels around 3 mg/L have been suggested as lethal from animal experiments. The toxicity of HCN is due to the binding to cytochrome oxidase in the mitochondria, and this precludes oxygen consumption in cells, leading to cytotoxic hypoxia. Additionally, cyanide ions react with methemoglobin to produce cyanomethemoglobin. However, the dynamics of HCN uptake and its related toxic effects did not receive the same degree of attention as for CO uptake: currently, no mathematical for HCN uptake has been widely adopted by the community. While data on human exposure effects is scarce, it is assumed that 50 ppm of HCN may be tolerated for about 1 h, but 130 ppm may be lethal in 30 min, and 180 ppm of HCN can lead to death in only 10 min [31].

**Nitric Oxide (NO)** passes very fast into the blood where it reacts with hemoglobin. It can form methaemoglobin that is a form of hemoglobin unable to bind to oxygen. In low oxygen conditions, it also binds to hemoglobin to form nitrosyl-hemoglobin (HbNO). These mechanisms have asphyxiant character by decreasing the oxygen transport capacity of the blood. It has been claimed that NO has 1500 times more affinity for hemoglobin than carbon monoxide. However, the dynamics and the parameters of these reactions are not totally understood [19].

**Nitrogen dioxide** at high concentrations is known to cause lung edema. According to ISO 13571, the incapacitating volume fraction of NO_2_ is 250 ppm [30]. This concentration is considered as the NO_2_ level that, if inhaled at any time, entirely limits the ability to escape from a hazard situation.

#### 3.2.2. Irritant Emissions

**Hydrochloric acid (HCl)** is an irritant gas that is extremely irritant to the eyes and the pulmonary system at 100 ppm, and it threatens life for short exposures of 1000 ppm or more. HCl is mostly emitted from PVC (and other chlorine-containing polymers) and its incapacitating power can be bigger than that of CO, but smaller than that of HCN.

**Hydrofluoric acid (HF)** and **hydrobromic acid (HBr):** Limited data exist on the toxic effects of these gases when inhaled. However, we may take as reference the values contained in the ISO13571 standard [30]. This standard is used in the estimation of the toxic potency of mixtures (see the section below) and it considers different reference values to weight the effects of the different constituents of the fumes, namely LC_50,HCl_ = 1000 ppm, LC_50,HF_ = 500 ppm, LC_50,HBr_ = 1000 ppm, where LC_50_ represents the gas concentration that is lethal for half of the exposed population during a time period (30 min).

**Sulphur Dioxide (SO_2_)** is an irritant gas that produces an increase airway resistance depending on the inhaled concentration. It can lead to pulmonary edema [35]. The incapacitating concentration according to ISO13571 is 150 ppm.

**Volatile Organic Compounds.** It is well-known that fire emissions may contain hundreds, if not thousands, of VOCs, but only a few have been considered from the point of view of fire toxicity. To mention just a couple, ISO13571 cites 30 ppm of acrolein and 250 ppm of formaldehyde as incapacitating values.

### 3.3. Combined Toxic Effects

The toxic potency of fire emissions can be estimated using several standards such as ISO 13344 and ISO TS-13571. These standards base their toxic potency calculations on the concentration of asphyxiant and irritant gases. The key compounds considered by these standards are CO_2_, CO, HCN, oxygen depletion, haloacids (HF, HBr, HCl), SO_2_, nitrogen oxides, formaldehyde, and acrolein. Despite evidence that NO plays a significant role in fire emissions, current ISO standards only consider NO_2_.

Different examples of models that take into account the combined effect of the toxic potency of mixtures with different toxicants have been presented. Fractional Effective Dose (FED) is obtained from the concentration of the components present in the mixture. Here, we present two examples of such models, (namely FED1 and FED2) [30,36]:

(3) $$\mathrm{FED}1=\frac{m\left[\mathrm{CO}\right]}{\left[{\mathrm{CO}}_{2}\right]-b}+\frac{21-\left[O_{2}\right]}{21-{\mathrm{LC}}_{50,O_{2}}}+\frac{\left[\mathrm{HCN}\right]}{{\mathrm{LC}}_{50,\mathrm{HCN}}}+\frac{\left[\mathrm{HCl}\right]}{{\mathrm{LC}}_{50,\mathrm{HCl}}}+\ldots$$

where FED is obtained from the concentration of the components and the parameters m and b. The parameters m and b model the increased ventilation caused by high concentrations of CO_2_. If [CO_2_] < 5%, m = −18 and b = 122,000 ppm. If [CO_2_] > 5%, m = 23 and b = −38,600 ppm. FED values depend on LC_50_ values. A value of FED = 1 is, hence, supposed to be lethal for half of the population after 30 min.

The additivity of effects has been empirically found in studies with rodents [37,38] and this model was also validated by Pauluhn [21].

An alternative formulation is [39]:

(4) $$\mathrm{FED}2=\left\{\frac{\left[\mathrm{CO}\right]}{{\mathrm{LC}}_{50,\mathrm{CO}}}+\frac{\left[\mathrm{HCN}\right]}{{\mathrm{LC}}_{50,\mathrm{HCN}}}+\frac{\left[\mathrm{HCl}\right]}{{\mathrm{LC}}_{50,\mathrm{HCl}}}+\ldots \right\}\cdot \left(1+\frac{\exp\left(0.14\left[{\mathrm{CO}}_{2}\right]\right)-1}{2}\right)\;+0.05\left[{\mathrm{CO}}_{2}\right]+\frac{21-\left[O_{2}\right]}{21-{\mathrm{LC}}_{50,O_{2}}}$$

It is important to remark that the values of LC_50_ are not the same for both models. This discrepancy is due to the diverse animal studies that were used to build the respective models. The values for FED2 are listed in Table 1, although FED values calculated with both equations may differ by approx. 30% [5].

When interpreting FED values, it is important to take into account the large variability in the resistance of people to the toxicological effects of fire fumes. In particular, children, the elderly, and people suffering from respiratory problems are more sensitive to toxicants. For this reason, the goal is maintaining fire conditions, when possible, in FED values substantially lower than 1. The literature mentions that at FED = 0.3, 11% of the population may suffer lethal consequences [5].

### 3.4. Toxicant Production Depending on Fire Scenarios and Burning Materials

The emission of fire effluents depends on the combustion conditions. In turn, combustion conditions depend on many factors such as ventilation, burning materials, room geometry, and overall fluid dynamics. We have already exposed that fire types can be divided according to their behavior and burning conditions: smoldering fires and open fires. Smoldering fires have been traditionally characterized by CO emissions, although many other volatiles appear, especially since the use of new building materials. Open fires do not pose a major threat in terms of intoxication danger in well-ventilated scenarios (*ϕ* < 1). However, in scenarios with limited ventilation, an open fire rapidly consumes available oxygen and it transits to under-ventilation (lean) conditions (*ϕ* > 1) that typically lead to the emission of toxic gases at high concentration levels. In this section, we focus the study on smoldering fires and open fires that already transited to lean conditions.

Since smoldering fires are cold fires (compared to flaming fires), the smoke is colder, and the buoyancy is also smaller. In consequence, the smoke disperses slowly in the full volume of the room, instead of rising straight to the ceiling, where smoke detectors are located. As a result, time to alarm can be longer for smoldering fires with respect to open fires.

**Figure 1:**
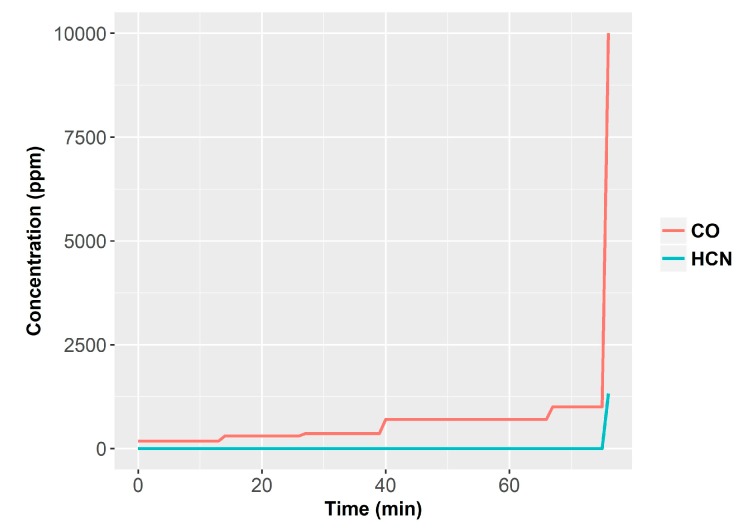
Example of evolution of CO and HCN for a smoldering fire (NIST tests) [40].

**Figure 2:**
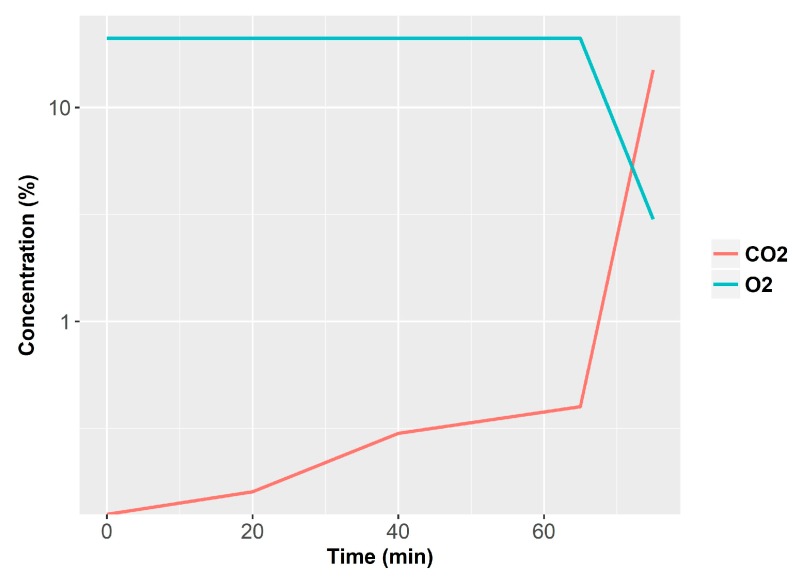
Example of the time evolution of O_2_ and CO_2_ during a smoldering fire [40].

**Figure 3:**
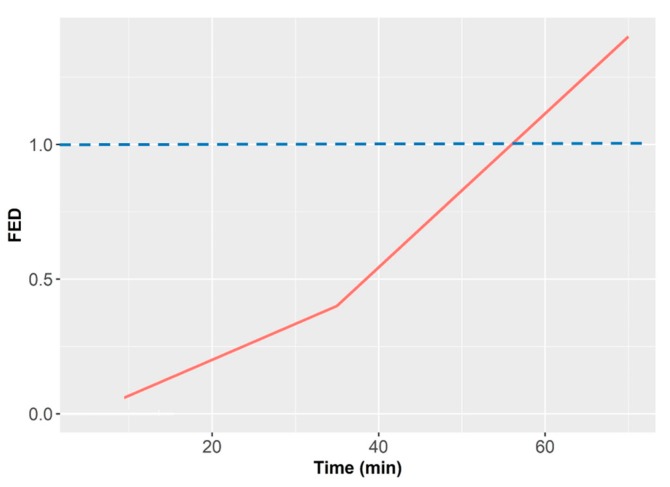
Time evolution of the toxic potency of the smoldering fire for the NIST test [40].

As example of smoldering fire and the release of volatiles, we can refer to a series of NIST tests fires performed with armchairs made of polyurethane foam with cotton fabrics [40,41]. The total mass was 5.7 kg, and the total volume of the room was 12 m^3^. The fire was initiated with two cigarettes placed over the chair. In this particular test, the fire run in smoldering conditions for 1 h before developing a flame. Figure 1, Figure 2 and Figure 3 show the progress of combustion products. We can observe how CO concentration builds up slowly in the room while the HCN presence only starts when flame conditions occur (Figure 1). On the other hand, O_2_ concentration also remains constant at 21% and lowers drastically only with the presence of open fire. Finally, the CO_2_ concentration increases slowly and finally it grows fast also in the case of open fire (Figure 2). We should remark that lethal conditions are attained due to the build-up of toxicants in the smoldering phase before flames appear. The Fractional Effective Dose reaches FED = 1 before the flame appears and before CO, HCN and CO_2_ concentration rise (Figure 3).

In a second recent example, SP Fire Research in Norway described a number of experiments based on smoldering fires [42]. The main goal of the report was to investigate if smoke detectors including CO sensors can alert occupants earlier than photoelectric smoke detectors. A secondary goal is to measure the concentration of toxic gases, mostly CO, during the development of the fire and determine if incapacity conditions are achieved before the photoelectric alarm triggers. The scenario they reproduce is a bedroom with a polyether foam mattress and cotton bed sheets. The room had a surface of 8.6 m^2^ and a total volume of 20.7 m^3^. The fire conditions were set to induce a smoldering fire on the mattress. Ten experiments were carried out, although one of the experiments was excluded because the fire developed into a flaming fire.

**Figure 4:**
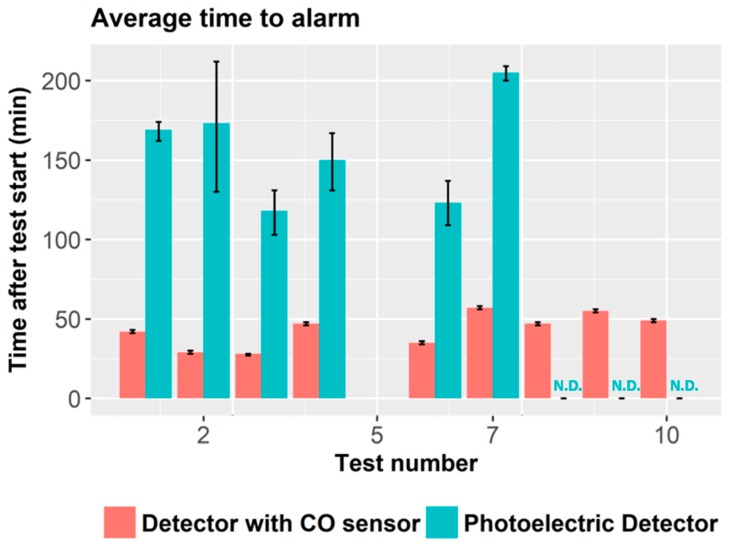
Time to alarm in smoldering fires in the SP Fire Research Experiment. Comparison between photoelectric detectors and multisensory including CO Electrochemical Cell [42]. Photoelectric detector combined with CO sensor always produced faster alarm signals, and it was able to detect all the test fires. Standalone photoelectric detector did not trigger the alarm for three of the fires (not detected [N.D.]). Experiment #5 was not considered in the study as the fire developed to open fire.

The main conclusions of the study were that the smoke detectors combined with CO sensors activated much faster than photoelectric detectors. The incapacitation limits due to CO intoxication were in some experiments achieved much before the alarm was triggered. This can, of course, have lethal consequences. In fact, in three of the experiments, the photoelectric detectors never triggered an alarm (three distinct brands and three different units for each brand were used). The time to alarm ranged typically from 2 to 3 h. Figure 4 compares the activation time between photoelectric detectors and detectors combined with CO sensors. Results show that detectors equipped with CO cells triggered the alarm signal for all the considered fires (whereas standalone photoelectric detector missed 3 of the performed fires) and produced alarm signals faster than photoelectric detectors.

The concentration of CO when the photoelectric triggered the alarm (evaluated at the mean time of the different units) ranged from 600 to 1500 ppm. The photoelectric detector triggered the alarm in six of the performed fires. In four of them, the integrated dose of CO had already reached incapacitation limits (calculated in windows of 30 min) when looking the CO levels at the mean time to alarm. Instead, the CO concentration ranged between 30 and 60 ppm when the combined detector triggered the alarm and, in no case the CO incapacitation limit was achieved thanks to the faster alarm response and the moderate concentration levels at the time of activated alarm (Table 2).

In summary, there are very clear evidences than in some fire scenarios (smoldering fires) the inclusion of chemical sensors (EC CO cells in this case) is the path to provide enough safety to building occupants. However, we would like to remark that commercial and standardized fire detectors do only consider the detection of CO as toxic gas. At this point, it is well established that many other toxicants may lead to lethal consequences (particularly but not only HCN). Since most of these toxic gases are not detected properly by CO electrochemical cells, current detectors cannot provide proper protection to building occupants. The widespread presence of polymers in building materials and also in consumer appliances and electronic products leads today to the appearance of new families of toxicants that need dedicated detection.

The use of polymers is widespread in furniture, but also in electrical appliances and consumer electronics that may overheat and be at the origin of fires. These materials start the emission of volatiles when overheated. This process is also known as thermal degradation or pyrolysis. Finally, the flammable gases emitted by the materials may burn themselves if sufficient heat is available. In fact, once initiated positive feedback, the process may be self-sustained. Additional terms that appear in the description of this process are polymer melting and charring. The gasification of polymer materials is a complicated process. When overheated, the non-volatile polymer breaks down into smaller molecules of many chemical species, each one characterized by a certain vapor pressure. In this way, the smaller and more volatile fragments will evaporate first, followed by bigger fragments. Eventually, bigger fragments may stay at the surface and suffer a posterior break down to smaller molecules. Typically, several residues appear that are mostly char and inorganic materials.

Polymers can be categorized according to many criteria. Chemical composition is the most suitable classification criteria when thermal degradation is under study. First, carbonaceous polymers only contain carbon and hydrogen atoms, being polyethylene and polypropylene two examples of carbonaceous polymers. There are also aromatic hydrocarbon polymers such as polystyrene. Some of these polymers appear blended with other polymers in commercial formulations. A second family of polymers is characterized by the presence of oxygen atoms. Among them, we encounter cellulosics, polyacrylics (like PMMA) and polyesters. Examples of polyesters are polyethylene terephthalate (PET), polycarbonates. Additional polymers with (H, C, O) are polyethers and polyacetals. The thermal decomposition of these polymers produces a large variety of alkanes and alkenes.

The third family of polymers is characterized by the addition of nitrogen (H, C, O, N). Examples are nylons, polyurethanes, polyacrylonitrile, and polyamides. For instance, thermal degradation of polyacrylonitrile starts between 250 and 350 °C and, among other products, it emits HCN and NH_3_ long before actual oxidation takes place.

Finally, polymers can contain other elements. Polymers containing chlorine are, for instance, polyvinyl chloride (PVC), polychloroprene or poly(vinylidene chloride). It has been reported that the emission of HCl from PVC starts at temperatures between 225 °C and 275 °C. Polymers like polytetrafluorethylene (PTFE), polyvinylidene fluoride (PVDF) or fluorinated ethylene polymers may also contain fluorine atoms [40]. Thermal decomposition of PTFE starts at temperatures around 475 °C and the main products emitted are CF_4_, HF, and hexafluoropropene.

Hence, a diversity of polymers that are nowadays used and found in home settings, and the different composition of these polymers results in a large variety of released volatiles at higher temperatures, when material degradation takes place.

## 4. Standards for Fire Detectors

Over the years several standards have been established worldwide to test the sensitivity and reliability of smoke fire detectors. One of the best well-known is EN54: “Fire Detection and Fire Alarm Systems” that specifies the conditions to be fulfilled by components and systems devoted to fire detection. This standard is mandatory in the European Union. It was created by the European Committee for Standardization (Comité Européen de Normalization—CEN) and it was also adopted by Latin American and Asian countries. For this paper, the most relevant section is EN-54: part 9: “Components of automatic fire detection systems. Methods to test the sensitivity to fire”. The EN-54 standard covers the requirements, test methods and performance criteria for point smoke detectors working under the principles of ionization, transmitted or scattered light. The same document states that for other types of fire detectors (e.g., fire detectors based on chemical sensors) the document must be considered only as guidance or inspiration. The standard covers the dimensions of the fire room, the position of the detectors in the room, and the required instrumentation that must be available for the tests. The document also details the procedure to perform the standard test fires. Table 3 lists the standard test fires described in the mentioned standard. The EN54 fires aim to prove that alarms have enough sensitivity to fire. The range of standard fires covers a diversity of aerosol types. It is important to note that not all fire detectors are suited to detect all fires. For instance, optical smoke detectors have poor sensitivity to liquid fires modeled by TF6, where smoke production is very limited. On the other extreme, flame detectors based on infrared or ultraviolet emissions are not suited for smoldering fire detection [43]. The principle of operation of the fire alarm selects the subset of standard fires to be used when testing the sensitivity of the detector. For more details on the technicalities and recommended procedure to implement those fires, the reader is referred to the EN-54 standard.

It is important to mention that there is a dedicated standard for smoke detectors aimed at residential use, namely EN 14604: “Smoke Alarm Devices”. In fact, EN54-7 and EN14604 share the methodology to select the most challenging conditions for smoke detectors, being test fires TF2 to TF5 the most relevant ones to be considered in dwellings.

It is also worth noting that, although the standard is described for smoke detectors, TF6 does not produce smoke or aerosols. Hence, the detection of this fire type requires different operation principles than those in conventional smoke detectors. In fact, the detection of TF6 fires requires multicriteria detectors [44] that usually include additional temperature sensors.

Additionally, the ISO-7240 standard “Fire detection and alarm systems” [45] is the international version of EN-54, and many parts are identical. There are, however, some differences because the working groups preparing the standards both are different. The definitions of the standard test fires are the same in both. Australia also adopted the ISO-7240 standard with only minor differences under the name AS-7240.

Other standards exist in the US, in particular, the NFPA-72: National Fire Alarm and Signaling Code [46]. This is a standard published by the National Fire Protection Association (NFPA) and recognized by the American National Standards Institute (ANSI). NFPA-72 focuses on the entire alarm system and on the electrical signals between fire alarm components. It does not cover the description of standard fires for fire sensitivity analysis. Hence, we will refer to the activity of Underwriters Laboratories (UL). UL is a global company headquartered in the US dedicated to safety. They have published more than 1500 standards in the area of safety [47], including standards relevant to the area of fire detection:

- UL217: “Standard for single and multiple station smoke alarms” [48]
- UL268: “Smoke detectors for fire alarm systems” [49]
- UL2034: “Standard for single and multiple station carbon monoxide alarms” [50]

UL217 and UL268 standards are described for different fire scenarios, although they share a lot of similarities. UL217 has the focus on residential smoke alarms, and UL268 focuses on smoke detectors connected to a central control unit. On the other hand, UL2034 is a standard for CO alarms to prevent intoxication due to inhalation. While the integration of CO and smoke alarms is very relevant, these products are not the focus of the UL2034 standard. The European equivalent of the UL2034 standard is EN50291 [51]. UL217/268 initially considered four flaming tests: namely paper fire (Test A), gasoline fire (Test C), polystyrene fire (Test D), and wood fire (Test B) plus a smoldering test consisting of ponderosa pine on a hotplate. Lately, the test set has been extended with flaming and smoldering versions of polyurethane foam [52].

The standard ISO7240-part 6: “Carbon Monoxide Fire Detectors using Electrochemical Cells” [45] is also very relevant for the consideration of fire detectors based on gas detection. Indeed, the mentioned standard acknowledges the importance of CO as a fire indicator. It also regulates the use of this type of systems for fire detection since this kind of products have been available since the late 90s. We have already mentioned the importance of CO as a toxic agent, mostly in slow, smoldering fires of carbon-based materials (wood, paper, etc.). The standard states that these detectors are important in scenarios where conventional smoke detectors are plagued with false alarms due to the large presence of dust, steam or other aerosols. The standard also warns users that detectors based solely on CO are not suitable for clean-burning liquids, PVC insulated cables, combustible metals, some self-oxidizing chemicals and non-carbonaceous materials. Additionally, since there are sources of CO that are not fire-related, some caution is necessary to take CO as an indicator of fire. This may happen particularly in scenarios that host CO sources like car parks. The standard sets the desired alarm level at 60 ppm of CO, and the standard also requires that no alarm should be given when CO concentration is lower than 25 ppm.

Alarms based on the ISO7240 standard are based on the definition of a single threshold value, whereas systems based on the UL2034 or EN50291 standards consider the accumulated dose of CO (interpreted in terms of COHb production). This results in the fact that alarms based on ISO7240 are even more sensitive to CO concentration than standalone CO detectors standardized under UL2034, or EN50291. For instance, at 60 ppm of CO (the alarm level for ISO7240), the UL2034 standard only mentions that the alarm should never be triggered before 28 min, and even not firing at this concentration is consistent with the standard.

**Figure 5:**
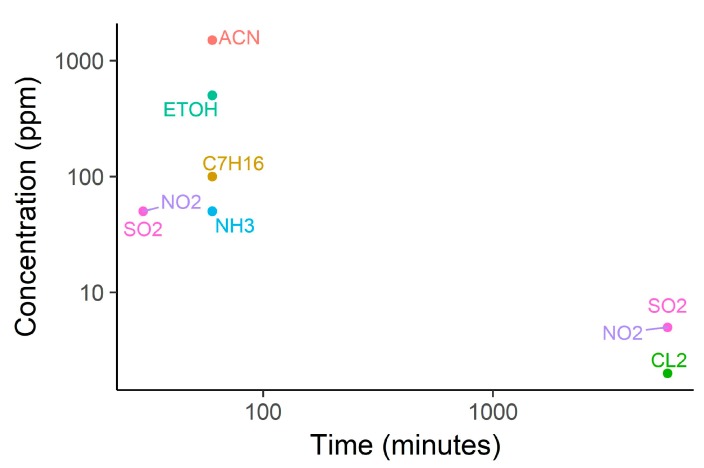
Concentration and exposure time of the different interferent gases that appear in the standard ISO7240. Note the log scale. Specifically, the concentrations and exposure times are: 5 ppm of NO_2_ at 96 h and 50 ppm at 30 min, 5 ppm of SO_2_ at 96 h and 50 ppm at 30 min, 2 ppm of Cl_2_ at 96 h, 50 ppm of NH_3_ at 1 h, 100 ppm of Heptane at 1 h, 500 ppm of Ethanol at 1 h and 1500 ppm of Acetone at 1 h.

A relevant feature of the ISO7240 standard, common to other standards in the area of chemical sensing, is the selection of a number of interfering chemicals that should not trigger the alarm (see Figure 5). The exposure of the detector to the presence of the interfering volatiles should not alter the compliance of the detector to the sensitivity tests. In this case, the standard selects nitrogen dioxide, sulfur dioxide, chlorine, ammonia, heptane, ethanol, and acetone. Additionally, the alarm should not be triggered in the presence of 5000 ppm of CO_2_. The robustness of detectors against interfering chemicals and nuisances is a must in detectors based on chemical sensors. The selection of the interfering chemicals and their concentration levels is always a matter of controversy and, obviously, it becomes dictated by the scenario where the alarm is to be installed.

The combination of smoke detectors, heat sensors and CO electrochemical cells to form a multicriteria fire alarm achieved higher commercial success than the use of standalone CO detectors for fire detection. Actually, this combination is standardized under ISO 7240-part 27: “Point-type fire detectors using scattered light, transmitted light or ionization smoke sensor, an electrochemical cell carbon monoxide sensor and a heat sensor”. In this case, the alarms are tested against standard test fires TF2, TF3, TF4, TF5, and TF8. The same chemical interfering volatiles considered in part 6 are also used for this embodiment of the fire alarm.

There are also country-specific standards covering electrochemical cells for fire detection. For instance, the Loss Prevention Standard LPS1274 covers “Testing procedures for the LPCB approval and listing of carbon monoxide/heat multisensory fire detectors using electrochemical cells”. This standard proposes to test the devices against TF2, TF3, TF4, and TF5, defined as in the EN-54 standard [53]. Similarly, we can encounter LPS1279: “Testing procedures for the LPCB approval and listing of point multisensor fire detectors using optical or ionization smoke sensors and electrochemical cell CO sensors and, optionally, heat sensors” [54]. In this case, TF8 is added as in the ISO standard [53].

As far as the authors know, there is no published standard regarding fire detection based exclusively on chemical sensor arrays. Obviously, ISO7240-part 6, can be taken as guidance. However, this document is tailored to CO electrochemical cells. The standardization of the fire sensitivity tests for fire detectors based on chemical sensor arrays could push forward the development of this type of detectors. In such a case, beyond standard test fires, attention should be paid to nuisances (causes of false alarms) as well. In the opinion of the authors, in addition to the selection of a number of interfering volatiles and their concentrations, nuisance scenarios have to be selected such that they ensure robust operation of these detectors in the selected scenario of use (domestic premises, buildings, commercial, etc.). This selection is not an easy task since the number of potential of sources of false alarms is large, especially when the sensor array includes sensor technologies with low selectivity, such as metal oxide sensors (MOX) or photo-ionization detectors (PID). As we will review in Section 6 and Section 7, while proposals already appeared in the literature, the community still needs to reach a consensus.

## 5. Gas Sensors for Combustion Products

In previous sections we have been reviewing the most significant components found in fire emissions from the point of view of their toxicity. The list contains CO, HCN, HCl, HF, HBr, NO, NO_2_, SO_2_ as inorganic asphyxiants and irritants, but one also needs to consider O_2_ depletion and CO_2_ levels, for their synergy with toxics: mostly because it results in an increased breathing rate. Additionally, it is well established that many VOC can also be emitted from fires, being acrolein and formaldehyde two of the most relevant examples. However, this only constitutes a short list and it is clear that the number of chemicals is enormous, it is practically impossible to have a chemical sensor dedicated to every single compound present in fire emissions. In this section, we will not refer to the possibility of using Fourier Transform Infrared (FTIR) analyzers for the simultaneous analysis of many emission compounds through the analysis of the absorption signature. We will refer only to the use of sensor components.

**Figure 6:**
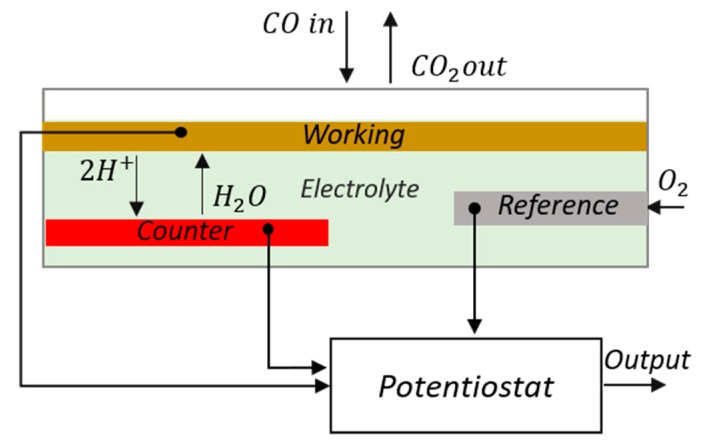
Nemoto NAP-505 three electrode CO sensing element.

The technology of choice for the analysis of most of the toxicants that appear in fire emissions is electrochemical cells. In fact, electrochemical cells are the standardized option when coupling carbon monoxide sensing to smoke detectors. Current standards do only refer to this technology and CO detection, disregarding other popular options such as metal oxide sensors with a larger number of target volatiles and cross-sensitivities [55,56,57]. Electrochemical sensors are based on REDOX reactions that produce an external current that is then measured. Typically a potentiostat circuitry in a three-electrode configuration is used. Figure 6 shows working principle for CO detection using electrochemical cells. There is a large variety of worldwide vendors offering sensors based on electrochemical cells for toxic gas detection. Some examples of commercially available sensors relevant for fire detection are given in Table 4, and additional vendors for these sensors follow in Table 5 [58]. We refer interested readers in the principle of operation of electrochemical cells to already published reviews [58,59].

Finally, we remark that some vendors offer different ranges of concentration for their products (the higher limits of the corresponding sensor ranges in Table 4 are only given for illustration purposes) and HCl, HBr and HF are sometimes detected with the same sensors designed for halogen acid detection.

**Figure 7:**
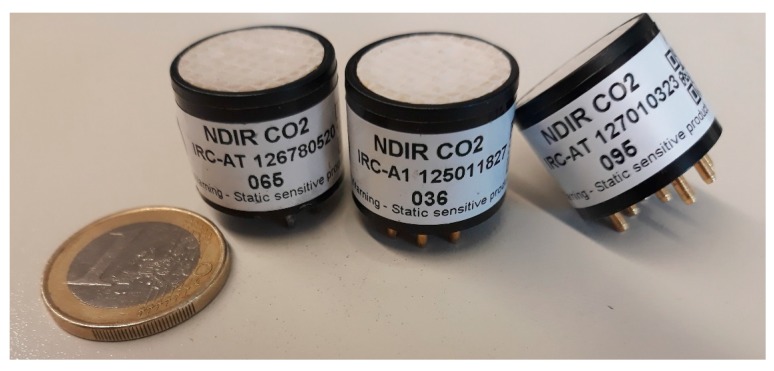
Alphasense offers miniature NDIR cells for CO_2_ detection in 20-mm diameter compact systems.

As already mentioned in Section 4, CO_2_ is a relevant gas in fire emissions. While it is not a direct toxicant, it increases the effects of others as we have seen in the models of toxic potency. The detection of CO_2_ at relevant concentrations in fire emissions is easily accomplished by miniature Non-Dispersive Infrared Cells (NDIR) provided by different vendors. The principle of operation relies on energy absorption in the infrared. CO_2_ absorbs at 2.7, 4.3 and 15 µm. NDIR sensors use infrared lamps, absorption chambers, wavelength filters and infrared detectors, although nowadays all the elements are integrated into a single system (Figure 7 shows examples of miniaturized compact NDIR systems). Typically, an absorption band and a reference band are used for compensation purposes. Additionally, temperature sensors are included to compensate for the influence of the operating temperature.

Finally, there are several VOCs that are also of interest but their selective detection at ppm level with simple sensors is not feasible today. Consequently, the detection of acrolein or formaldehyde, for example, should be targeted with non-selective sensors. Two main technologies are available nowadays. On the one hand, we can find photo-ionization detectors (PIDs) that are based on the ionization of target molecules by a UV lamp. Different volatile compounds have different efficiency regarding the ionization process but, if the molecules can be ionized by the energy of the lamp (typically from 8.4 eV to 11.8 eV), the detector will give a response. Thus, PIDs are considered as non-selective sensors since a weighted overall VOC reading is obtained. The advantage of PID sensors is that they achieve very low detection limits (in the order of ppb) but at the expense of being also sensitive to harmless chemicals that may appear as nuisance during normal daily activities (cleaning products, perfumes, etc.). Metal oxide gas sensors (MOX) are a more robust alternative, but this choice is also plagued with problems of very poor selectivity. On the one hand, the broad response of MOX is beneficial to detect a large number of combustion products and provide additional safety to a building’s occupants, but, on the other hand, the non-selectivity makes this technology more prone to false alarms. To gain some selectivity for fire signatures, arrays of MOX sensors or temperature modulation strategies must be used. Consequently, the use of these devices for fire detection should necessarily include some computational intelligence that is able to differentiate fire signatures from nuisances. Therefore, only after a data processing step one can obtain reliable fire detection. This will be reviewed in the section devoted to the algorithms (Section 6 and Section 7).

## 6. Fire Detectors Incorporating Chemical Sensors

### 6.1. Decision Tree and Hard Rules

Traditionally, fire alarm systems based on smoke detection make use of a single threshold value to define the fire region. This region can be defined more accurately by taking into account readings from other sensors and building a set of thresholds (or rules) that incorporates the multiple sensor signals.

**Figure 8:**
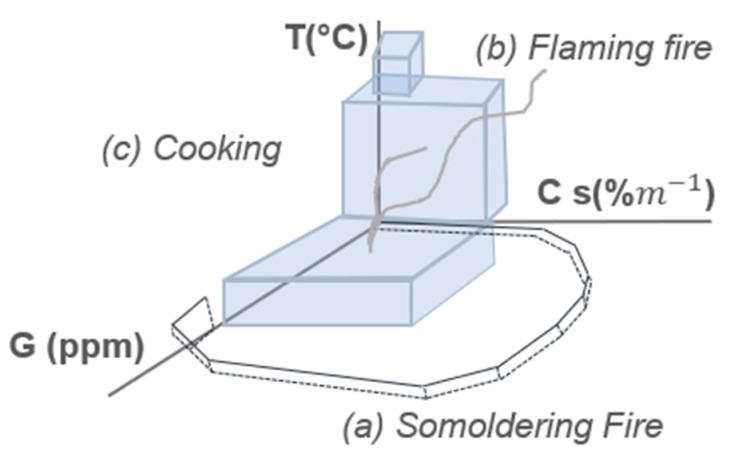
Defined fire regions when smoke detector (% obscuration per meter) is coupled with temperature (°C) and CO (ppm) measurements. Additional information provided from other sensors help to define more specific fire regions than when only smoke detector is used. The threshold planes were set to discriminate smoldering and flame fires (sensor signals **a**,**b**) from cooking (sensor signal **c**). During cooking, at the beginning, only temperature increases. As the food is becoming charred, smoke density increases, but no fire alarm is triggered as the signal stays within the defined non-fire region. Adapted from [8].

In the early nineties, Ishii et al. presented an approach based on hard rules and a smoke sensor coupled with a thermocouple and a semiconductor CO sensor [8]. The multi-sensor system was placed in a 6.7 × 4.3 × 2.5 m^3^ room in which smoldering fire (wood), flaming fire (n-heptane) and cooking activities (grilled fish) were performed. Based on the instantaneous reading of the three sensors, the authors defined specific regions in the sensor space to limit the fire region. Figure 8 shows the defined regions and their complexity. Based on the set of rules, fire alarm is only triggered when the acquired point falls outside the volume enclosed by the different planes. As a result, cooking activity did not trigger fire alarm, although smoke density showed response to this activity, which may have reached obscuration threshold limit defined for smoke detectors.

The proposed set of rules, though, is very specific to the experimental setup and tested fire/nuisance scenarios. In order to provide a more general model, the authors proposed a method that uses dynamic features and relies on sensor correlation. In particular, using a similar experimental setup, they found out that heat release and volatile release come together in the performed fire test (metal chair with polyurethane cushion and polyolefin fabric). This sensor correlation was significantly smaller in the tested nuisance scenario (cooking). They proposed, thereby, to use the correlation between heat release and volatile release (and its rate of change) to detect fires. However, unfortunately, authors did not validate this approach with unseen measurements. Moreover, smoldering fires with very slow combustion process may initiate heat release significantly after volatile release and, therefore, the proposed signal correlation may not be a good indicator to predict slow smoldering fires.

In the mid-nineties, research teams from the Department of Fire Protection Engineering and the Department of Chemical Engineering at the University of Maryland (College Park, MD, USA) joined efforts to detect fire situations using a variety of sensors. Initially, researchers performed experiments in a small-scale setup (see Section 7), in which only chemical sensors were used and samples were introduced using an atomizer. In this section, we will focus their efforts on a continuation work where the sensor system was placed in a larger experimental setup (3.6 × 3.6 × 2.4 m^3^), and it included gas sensors and light obscuration sensing.

Specifically, the system integrated TGS880 and TGS822 MOX gas sensors (Figaro, Japan), CO (PIR 2000, range 0–1% Horiba, Irvine, CA, USA), CO_2_ (Horiba PIR 2000, range 0–5%), O_2_ (540A, range 0–20.95%, Servomex, Belgium) sensors, a temperature sensor (thermocouple) and light obscuration detector (OSD-100-5T-BNC, Centronic, UK). Moreover, for comparison purposes, the setup was equipped with two commercial smoke detectors (one photoelectric and one ionization) [60,61]. They performed 87 tests, including 34 flame fires, 16 smoldering fires and 37 nuisances.

The dimension of signals captured with the two MOX sensors, CO and CO_2_ sensors and temperature and light sensors was reduced to three dimensions by means of Principal Component Analysis (PCA). Therefore, the dimension of the space was shrunk from six to three, while the three principal components captured 76% of the variance of the original data. They built hard rules on this new space to classify flame fires, smoldering fires or nuisances. The scores were used to define the boundaries of each region as follows:

**If**: PC3 > 5: **Flaming fire**.
**If** −8 < PC2 < 0: **Smoldering fire**.
**Else**: **Nuisance**.

They compared the performance of the chemical system with a commercially available smoke detector. While commercial detector did not trigger the alarm for 16 of the 50 tested fire conditions, this number was reduced to only two for the multisensory system based on dimension reduction and hard rules. Proposed method also outperformed commercial system in response time, as, by average, flaming fires were detected 45 s faster and smoldering fires were detected 245 s faster, which represented a time reduction of 57% and 30% respectively. However, the system with gas sensors was very sensitive to nuisances as it produced false alarms for 10 out of 37 conditions (10 nuisances were wrongly identified as smoldering fire), while the smoke detector only showed 4 false alarms [62].

False alarm ratio was improved, at the cost of reducing sensitivity to smoldering fires, when the authors revisited the dataset and considered a new set of sensors. In particular, the system included two MOX sensors, CO and CO_2_ sensors and the temperature sensor [62]. In other words, the photocell was removed from the array of sensors. Using hard rules based on the sensor signals the authors could classify smoldering fires, flame fires, nuisance cases, and background. The rules were defined as follows:

**If**: CO_2_ > 210 ppm **or** T > 105 F: **Flaming fire**.
 **Elseif**: V_TGS822_ > 0.9 V **and** V_TGS880_ > 0.15 V: **Nuisance**.
  **Elseif**: CO > 17 ppm **and** CO_2_ > 22 ppm **and** V_TGS822_ > 0.27 V: **Smoldering fire**.
 **Else**: **Background**.

where V_TGS8xx_ denotes acquired voltage from the corresponding MOX sensor conditioning circuit.

Table 6 shows the confusion matrices for the commercial smoke detector, and the two considered multi-sensor arrays with the corresponding decision models. The systems that included chemical sensing outperformed smoke detector in terms of sensitivity to fires. Similarly, the system with the light obscuration sensor showed higher sensitivity to smoldering fires than when the light sensor was removed. However, whether this is due to the information provided by the light sensor or due to the employed decision algorithm remained unexplored. On the other hand, chemical systems showed a higher rate of false alarms than the smoke detector. Actually, as all considered methods rely ultimately on the definition of thresholds, sensitivity and specificity could be adjusted by tuning the corresponding thresholds.

In another work, the same research group explored fire sensitivity and nuisance immunity using another multi-sensor system and different hard rules [64]. Specifically, they exposed a photoelectric smoke detector, ionization smoke detector, CO sensor and thermocouple to 32 fire tests (smoldering and flaming) and 11 nuisance (cooking tests, smoking and candle) scenarios. Captured signals were filtered to reduce noise and get rid of data spikes. Instantaneous values and rate of rise for each of the sensors were considered.

Authors proposed nine different hard rules using different combinations of sensors and features. Resulting sensitivity and specificity were evaluated individually for each set of rules, and they were compared to thresholded smoke detectors. Results indicated that the rule involving the rate of temperature rise, CO concentration, and smoke detection (using ionization detector) provided the best immunity to false alarms and fire sensitivity. In particular, the selected rule was as follows:

**If**: (Rate of T > 0.2 °C/s) **or** (CO > 17 ppm) **or** (Ion > 0.15% obs/m): **Fire**.
  Else: **Background**.

The authors concluded that rules that included CO measurements resulted in faster detection of smoldering fires than smoke detectors. Similarly, the rate of temperature rise resulted in faster fire detection, or at least, similar, than smoke detectors. Authors also proposed several rules to define fire/non-fire regions after PCA was applied to data. However, authors did not find any significant improvement after defining ellipses in the lower-dimension space. Authors attributed the similar performance of the rules defined directly in the sensor space with the rules defined after the PCA to the limited number of sensors which is not large enough to flourish the benefits of dimensionality reduction.

In summary, the research efforts carried out by the Department of Fire Protection Engineering and the University of Maryland showed that simple hard rules could be defined such that fire and nuisance situations can be discriminated. They also showed that dimensionality reduction could be performed before the definition of the decision rules. When compared to smoke detectors, chemical-based fire detectors showed improved sensitivity, although it came at expenses of higher false positive rate. The remaining challenge is keeping high sensitivity while specificity remains at acceptable levels.

Chen et al. proposed a system that combined smoke detector with carbon monoxide and carbon dioxide measurements [65]. They compared the performance of the multi-sensor system with the performance of only the smoke detector. The smoke detector was based on light scattering and, when operating alone, it triggered a fire alarm when the threshold of 15% obs/m was reached. CO and CO_2_ detection were performed by means of a diode laser-based absorption spectrometer, which was composed of a laser, InGaAs diodes and reference and measurement cells.

The proposed algorithm for the multi-sensor system was based on dynamic features, specifically, the rate of change of the smoke, CO, and CO_2_ signals. Then, a decision tree was built to output, continuously, fire/non-fire prediction. Briefly, fire was only predicted when smoke rate of rise was higher than a threshold and the rate of rise of CO or (non-exclusive) the rate of rise of CO_2_ were higher than the corresponding thresholds. The authors explored two methods to estimate the signals’ rate of increase. First, the rate of rise was estimated fitting a linear function to the captured data points using 10-s time windows. The second method included a moving average filter before the linear fit was computed. The thresholds were adjusted for each volatile and method, resulting in the following rules for the first and the second methods respectively:

**If**: (Rate of V_smoke_ > 1 mV/s) **and** [(Rate of CO > 0.15 ppm/s) **or** (Rate of CO_2_ > 25 ppms/s)]: **Fire**.
  Else: **Non-fire**.
**If**: (Rate of V_smoke_ > 1 mV/s) **and** [(Rate of CO > 0.05 ppm/s) **or** (Rate of CO_2_ > 8 ppms/s)]: **Fire**.
  Else: **Non-fire**.

where V_smoke_ represents the voltage captured from the output of the smoke detector. The mentioned algorithm was patented by the authors [66].

The authors tested their approach using a collected dataset that included a total of 30 fires (smoldering and flame) performed in a 2.2 × 1.4 × 4 m^3^ unventilated room. Smoldering fires included HDPE beads, PVC clad wire, mixed fabrics (with different ignition methods) and green canvas. Flame fires included heptane, toluene, methanol and mixed plastics. Two or three repetitions were carried out for each fire type. Authors also tested immunity to false alarms. In particular, they tested nuisances that may be present in aircrafts. Specifically, tested nuisances included dry ice, insecticide bomb (aerosol), halon, water, methanol, ethanol, acetone, and ammonia.

Results indicated that there is no significant difference between the two methods proposed to compute the signal derivatives, and no false alarms were detected throughout the tests. However, the multi-sensor system showed better sensitivity to fire than the smoke detector. Due to the small amount of smoke released by heptane, methanol, PVC wire and mixed fabrics, smoke detector did not trigger fire alarm for these four types of fire. The multi-sensor system only missed methanol fire. However, the authors adapted the rules such that fire is predicted when two of the three rate of rise features exceeded the corresponding threshold. With the new formulation, the multi-sensor system was able to detect methanol fire as well. Moreover, multi-sensor system also showed improved detection time, reducing, for example, detection time of HDPE bead fire from 616 s to 320 s.

The authors showed that defining rules based on the rate of change of the signals may be beneficial, as these dynamic features overcome issues with baseline shifts and may detect changes faster. Finally, the outstanding sensitivity and robustness to false alarms of the multi-sensor system may be due to the specificity of the employed chemical gas sensors. The immunity to false alarms may not be found when using less-costly, broad-response gas sensors.

Gottuk et al. presented a system that combined smoke detectors with CO detection using an electrochemical gas cell [67]. The authors performed fires and nuisances in a 49 m^3^ room. Large variety of flame and smoldering fires (heptane, alcohol, gasoline, flaming polyurethane, smoldering polyurethane, cardboard, cotton fabric, flaming cotton wick, smoldering cotton wick, cotton batting, upholstery fabric, PVC cable, smoldering wood at different temperatures) and nuisances (Wesson oil, toast, melting cheese, bacon, propane burner, kerosene heater, cigarette smoke, people smoking, water steam) were induced in the room, with different number of repetitions each scenario. Two smoke detector systems (ionization and photoelectric), along with gas sensors were installed in the test room. 

The authors set the detection threshold of smoke detectors to 4.52% obs/m for the ionization detector and 6.72% obs/m for the photoelectric smoke detector. Results confirmed that ionization detectors show better sensitivity to flaming fires, whereas photoelectric detectors show better performance for smoldering fire detection. 

**Figure 9:**
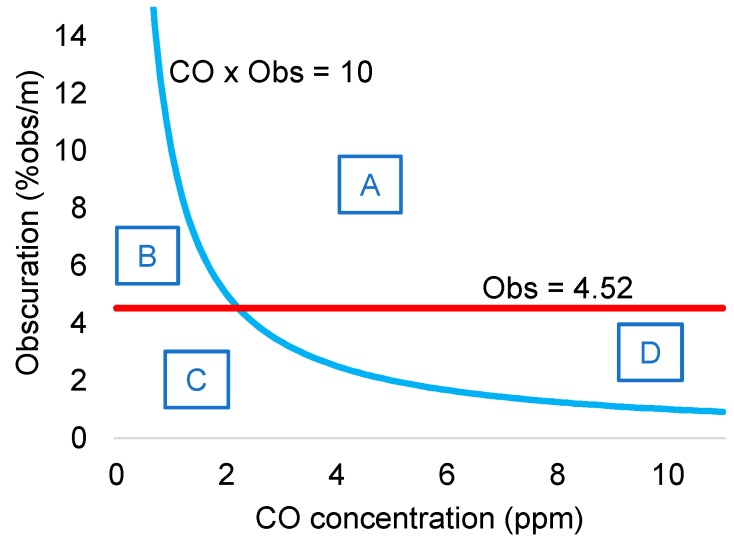
The coupling of CO measurement to light obscuration detector allows the definition of more specific fire/non-fire regions. Light obscuration detectors traditionally set fire alarm when the signal reaches a certain threshold (4.52% obs/m in this example, red line). The function Obs = 10/[CO] (blue line) defines a new boundary for fire/non-fire regions. Region A: Multi-sensor and smoke detectors output fire alarm. Region B: Only smoke detector outputs fire alarm. It is assumed that high obscuration signal and low CO concentration corresponds to nuisance scenario (water steam, dust, etc.). Region D: Only multi-sensor system outputs fire alarm. High CO concentration levels may come from incomplete combustion processes. Region C: No alarm region. Adapted from [67].

The proposed multi-sensor algorithm for fire detection was based on the readings from the ionization fire detector and the CO sensor. The authors developed a simple rule that takes into account the readings from both sensors such that high concentrations of CO also triggered fire alarm. In particular, the criteria was as follows: the alarm was triggered when the product of the ionization detector output (% obs/m) times the CO sensor reading (in ppm) was greater than 10 (% obs/m)(ppm). By coupling the CO sensor to the ionization smoke detector, boundaries of fire/non-fire regions could be defined, as shown in Figure 9.

The multi-sensor system was compared to traditional smoke detectors. Despite the simplicity of the proposed rule, the multi-sensor system detected 42 out of 53 fire tests, while ionization and photoelectric detectors detected 25 and 29 of the tested fires, respectively. Briefly, the multi-sensor system detects the union of the set of fires that are detected by the ionization and the photoelectric detectors, except for some smoldering wood (at lower temperature) and PVC cable, which can be detected by photoelectric detector and did not trigger alarm for the ionization smoke + CO detector. 

Immunity to false alarms was also improved with CO measurement. For example, water steam increased obscuration measure and triggered smoke detector alarms, but it did not increase CO sensor readings, which prevented triggering fire alarm for the multi-sensor system. Photoelectric and ionization showed false alarm to 17 and nine of the 27 tested nuisances. Multi-sensor system only triggered false alarm in six of the nuisance scenarios. Moreover, time response was also improved. Ionization detector coupled to CO sensor showed faster response time than ionization detector alone, except for heptane and polyurethane fires.

The authors showed that adding CO measurements to light obscuration sensor can improve both fire sensitivity and false alarm immunity. Simple hard rules can successfully process sensor signals. However, the authors already discussed a limitation of the proposed rule as its asymptotic nature makes it necessary very high levels of CO concentration (or smoke) if smoke (or CO concentration) levels are very low. This rule will delay the detection of fires that, for instance, generate small CO concentration. The authors proposed adding additional rules to cut the asymptotic behavior in its limits.

All in all, hard decision rules have been explored recurrently over the years. The popularity of this choice is probably due to the classic operation of smoke-detectors that rely on signal thresholds. The natural path is, hence, reshaping fire regions defined with light obscuration thresholds to obtain more accurate fire regions that incorporate additional information from chemical gas sensors. On the bright side, hard rules are considered as “white boxes” as they are easy to interpret [68]. Acquired knowledge of the system behavior is translated to a readable set of rules.

On the downside, decision rules may become too complex when many different nuisances are considered, as each scenario may require its own set of conditions to be excluded from the fire region. Also, and most significantly, hard rules depend heavily on the presented dataset. This is usually not-desired as one aims at building models robust to noise and able to generalize to new data or new experimental conditions (room size and geometry, fire types, nuisances, etc.). One limitation that we found in the literature is the fact that generalization to other experimental conditions is not explored. To what extent defined rules are valid when the system is placed in a different room, under different ventilation conditions or when the sensors are at different distances from the fire source remained, mostly, unexplored.

Dynamic features were also proposed to improve the accuracy and the generalization ability of the models. For example, it was found that rate of rise of CO and CO_2_ concentration levels can improve the ability of the system to discriminate between fire and nuisance scenarios. In reference [69], only one nuisance showed CO_2_ increase rate higher than 0.1 ppm/s, and only two nuisances induced CO increase rate higher than 0.025 ppm/s. Although CO_2_ was found to increase at high rates during fire, it also does so when the room is occupied by individuals (the presence of people in a non-ventilated room can induce CO_2_ increase rates as high as 0.5 ppm/s). Therefore, CO rate of rise was suggested over CO_2_ rate of rise to discriminate fire from nuisances.

Also, using dynamic features, such as rate of rise, becomes beneficial as these features are insensitive to baseline shifts and may provide faster responses. For example, derivative features were shown to change faster than the mean of the signal computed in the same time window [65].

Similar to static features, thresholds for dynamic features may be also specific to room size or geometry. However, experiments in two test rooms suggested that room effects can be incorporated to the model by including (and adjusting) rate of rise thresholds in the algorithms [63].

Finally, approaches based on linear data transformation (PCA) have been proposed to define hard rules in the transformed sensor space. These rules may be intricate and complex in the original space, but they may become simple in the new space. Moreover, if enough repetitions are included in the original data matrix, the new data projection can find the *mean* direction for each fire/nuisance type and reject inherent variability for each scenario [64].

Hard decision rules have been proved to provide good prediction ability when tested under the same conditions than the calibration conditions. However, other classification algorithms that usually show lower generalization error [68] have also been explored for reliable fire detection. 

### 6.2. Neural Networks

In the early 1990’s, Okayama studied the use of neural networks to assess the risk of fire using a variety of sensors [70]. He adapted the configuration of the neural network to address three different tasks, using different sensor ensembles and sensor features for each task.

First, a three-layer neural network with three input neurons, five hidden neurons and three output neurons was used to output three fire indicators. Three sensors (temperature, carbon monoxide sensor, photoelectric smoke sensor) were considered to feed the input layer of the network. Static features for CO and smoke sensors were extracted, whereas dynamic feature (rate of rise) was extracted from the temperature sensor. Additionally, to extract the corresponding features sensor readings were normalized such that the ranges 0–20% obs/m, 1–100 ppm of CO, and 0–10 °C/min were mapped to the interval 0–1. The output of the network was associated with three indicators (fire probability, fire risk and smoldering fire probability), which were also set in the range of 0–1. The neural network was trained using 12 fire patterns. 

In the second task, only the photoelectric smoke sensor was used. Two features were extracted from the sensor signal: instant value and rate of rise. The features were also normalized to the range 0–1, corresponding to 0–20% obs/m and 0–20% obs/m per minute, respectively. The architecture of the network consisted of two input, four hidden, and two output neurons. The relevant output neuron was associated with fire probability and 18 fire patterns were presented to train the network weights.

Similar to the second task, the third task considered only the photoelectric sensor, but the dynamic feature was changed. In particular, the two extracted features were the instantaneous sensor reading and the time duration (normalized to 0–1) since the sensor signal exceeded a defined threshold. The network consisted of two input neurons, four hidden neurons, and one output neuron (that accounted for fire probability). The network was trained with 10 patterns. Finally, task 3 was extended to consider ventilation conditions. Ventilation was incorporated to the neural network as a third digital input that took 0/1 for ventilation on/off.

After the mentioned neural networks were trained, output values provided by the model showed acceptable correlations with the defined values, also when chemical sensors were combined with smoke detectors. Unfortunately, different measurements were used to train the different models, making not possible the comparison between the considered tasks. Moreover, very few details on the experimental protocol are presented in the original work, the time at which the vector of features was extracted to feed the neural network was not specified, or details on the criteria to quantify the output indicators were omitted, which represent the alarm signals. Nevertheless, results presented by Okayama were encouraging as, although the simplicity of the neural network, the model could assign a probability to the presented measurements. He also considered dynamic features, showing that there is relevant information in the temporal response of the signals. Actually, he envisioned that further work should consider models that are able to process time-series directly. 

In a following work, Okayama and Sasaki considered nuisance scenarios, which were omitted in Okayama’s previous work. They coupled a MOX gas sensor to a smoke detector to discriminate fire from nuisances using a neural network (four input neurons, four hidden neurons, one output neuron) [71]. The sensors were exposed to sixteen measurements that included fire repetitions (beechwood smoldering fire at 2 m or 3 m from the sensors) and non-fire situations (smoking, cooking, coffee aroma, background). Four features were considered to feed the three-layer neural network: normalized sensor level and normalized rate of change per minute, for each sensor. The output neuron was associated to fire probability. For training the network, fire probability was manually assigned in the range 0–1 according to the distance of the sensors to the fire source or the type of nuisance.

Unlike their initial work, the neural network processed all the captured signals continuously. As a result, fire probability was provided as a function of time. Results showed that the system was able to output fire probability continuously, providing reasonable values as smoldering fires were being developed. However, the model showed difficulties to reject nuisances (mainly cooking activities). This shortcoming was attributed to air turbulence that took place in the test room (270 m^3^) that limited the accuracy of the classifier [71,72].

In a similar work also using neural network, Okayama studied the feasibility of fire detection using only chemical sensors (see Section 7, reference [73]).

In order to reduce fire detection time and increase the reliability of fire detectors, Derbel integrated three metal-oxide gas sensors with a commercially available optical (light-scattering) fire detector and a temperature sensor [4]. Specifically, the gas sensors were selected for carbon monoxide, hydrogen, and ammonia detection.

The system was exposed to flaming fires (TF1, TF4, TF5) and smoldering fires (TF2, TF3) inspired by the EN54 standard, a non-standard fire (cable fire) and two nuisance scenarios (disco-fog generated with a commercial fog machine, and cigarette-smoke using a force pump that regulated the burning process).

In order to build a model to detect fires, different dynamic features were tested. First, a moving window and FFT transformation provided features from the sensors’ signals. Second, feature extraction was performed by means of scaling the quadratic mean value of the signals, and then a back-propagation neural network was used to output the prediction. In both cases, results indicated that incorporating chemical and temperature sensors to the optical fire detector provided faster alarm signals in a more reliable manner (unlike the optical fire detector, the multisensory system did not show false alarms for cigarette smoke and disco-fog).

However, since no repetitions were acquired, models were trained and tested using features of the same measurements. Features of sensor signals corresponding to the same measurement were distributed in train and test. TF1, TF2, TF3, TF5, cable fire and cigarette smoke appear both in training and test, and only disco-fog and TF4 were left completely for test. Hence, training and test vectors are not completely independent. This questionable dataset partitioning yielded, most likely, to overfitting and overoptimistic results.

Finally, to what extent the performance increase of the system is due to the integration of the temperature sensor or the chemical sensors was not explored. This would provide very meaningful insights for the design of chemical-based fire detection systems.

Neural networks have shown good performance for fire prediction. However, more elaborate networks have been presented to account for the prior probability distribution function, such as Probabilistic Neural networks. Taking into account prior probability seems critical in fire prediction, as one expects the system in rest state for most of the time.

### 6.3. Probabilistic Neural Network

A remarkable work in fire detection was published by Rose-Pehrsson et al. (Naval Research Laboratory) in 2000 [74]. They studied the response of different sensor technologies to 24 different types of fire and 12 nuisances (see Table 7 for the complete set of fire/nuisance scenarios considered in the study). Several repetitions of the scenarios were performed in a 96 m^3^ test compartment, for a total of 240 events (120 background recordings, 82 fires and 38 nuisance sources). To the best of our knowledge, the considered dataset represents the largest dataset, with the largest variety of fire types and nuisance scenarios, collected for fire detection with chemical gas sensors. The large variety of fire and nuisance sources enabled a thorough study on fire detection sensitivity and system reliability. Moreover, the authors also placed a large number of sensors in the measuring compartment. The variety of sensing technologies and the benchmark measurements performed with commercial smoke detectors allowed to achieve another relevant goal of their work: the study of sensor similarities and the selection of an optimal subset of sensors for reliable fire detection.

In particular, the authors placed 20 sensors of different types in the measuring compartment. A variety of chemical gas sensors was installed to target various combustion products. Chemical sensors included carbon monoxide (at two concentration ranges), oxygen, hydrogen, hydrogen chloride, hydrogen cyanide, hydrogen sulfide, sulfur dioxide, nitric oxide and nitrogen dioxide electrochemical cells, a NDIR for CO_2_ and a MOX for hydrocarbon detection. Commercially available smoke detection systems (ionization and photoelectric) and an optical density meter system were also included to obtain reference measurements, and temperature and humidity were monitored during the measurements as well.

The sensitivity to fire detection and the immunity to nuisance sources of photoelectric and ionization fire detectors were used to benchmark the system that incorporated gas sensors. Conventional alarms were triggered when signals reached different obscuration thresholds. In particular, three thresholds were tested for each smoke detector. First, alarm thresholds were set to 4.2% obs/m for ionization and 11.0% obs/m for photoelectric detectors, which correspond to typical alarm thresholds. Minimum alarm level allowed by the UL 268 Standard (1.63% obs/m) and half of it (0.82% obs/m) were also tested as alarm thresholds. Using a total of 120 events (82 fires and 38 nuisances), confusion matrices for each detector type and threshold values were computed. 

Results with smoke detectors showed that, at lower alarm levels, systems showed high sensitivity to fires, but low immunity to nuisances. At lower alarm levels, 73% of the fires were correctly detected by the photoelectric detector, but false alarm ratio was as high as 47%. Oppositely, at the higher alarm level, the system could detect only 38% of the fires, while 82% of the nuisances were rejected. Similar behavior was observed with the ionization detector. When background measurements were also included, best overall classification ratio were obtained at lower threshold alarm levels (83% and 88% for photoelectric and ionization detectors respectively).

The obtained classification ratio values served as a benchmark to compare the performance of gas sensor-based fire detection systems. The authors developed a pattern recognition algorithm for fire detection based on probabilistic neural networks (PNN). All gas sensor signals were filtered with Savitzky-Golay routine to reduce noise. Only steady-state features were considered, which were extracted at discrete times defined by reference photoelectric detector. Finally, before training PNN, matrices were scaled to zero-mean and unit-variance. The authors followed a leave-one-out cross-validation strategy, i.e., they sequentially trained all but one observation and predicted the class of the sample that was left out. This procedure was repeated until all the measurements were set aside for test.

Best results were obtained with a subset of five sensors: O_2_ (model 6C, City Technology, Portsmouth, UK), H_2_S (model TC4A-1A, City Technology), RH (model HX93, Omega, Stamford, CT, USA) ionization smoke detector (model 4098-9716, Simplex, Westminster, MA, USA) and photoelectric smoke detector (model 4098-9701, Simplex). With this array, 98% of correct classification was achieved.

The authors concluded that smoke detectors are important for the detection of fires. Results showed that systems including at least one smoke detector had higher sensitivity to fire. However, results indicated that gas sensors provide additional useful information for the discrimination of nuisances and early fire detection. Actually, nuisance rejection could be improved up to 25% when CO_2_, O_2_, CO, hydrocarbons, temperature and NO sensors were combined with smoke detectors at the lower threshold level.

In a continuation of their work, the authors demonstrated the flexibility of the PNN algorithm [75]. Using a subset of sensors (photoelectric smoke detector, ionization smoke detector, CO and CO_2_ sensors), they adjusted probability density function for each class. As a result, they could define the boundaries for each class. When the threshold was set to 100%, no false alarms were found, but only 60% of fires were successfully detected. As the threshold was lowered, fire detection ratio increased, at the cost of increasing false alarm ratio as well. By plotting the sensitivity and false alarm rate in a Receiver Operator Curve (ROC), the authors could select the threshold (85%) that provided similar detection rates than reference smoke detectors. However, false alarm rate was greatly reduced. At the selected threshold, the system detected 78% of the fires and less than 20% of the nuisances produced false alarm. This result clearly improved performance of reference smoke detection systems, as they showed 66.7% and 74.1% of sensitivity and 66.3% and 41.7% of false alarm ratios for ionization and photoelectric systems, respectively. Results, therefore, confirmed their previous findings that suggested that combining gas sensors with smoke detectors helps to reduce false alarm rates.

All in all, work from Rose-Pehrsson et al. confirmed the feasibility of chemical gas sensors for fire detection and that gas sensors can improve false alarm immunity. The work is particularly valuable as it relies on an extensive dataset that included 24 fire types and 12 nuisances. By collecting such dataset, the authors ensured the generalization of their approach, which sometimes is overlooked by other works due to the cost of the experimental setups and data acquisition. They also explored different sensors targeting various combustion products and proposed a reduced set of sensors for fire detection. A final decision on the sensing technologies should be taken according to target specifications and also other considerations such as system cost, time stability, calibration cost, power requirements, size, and others.

Finally, the authors also remarked that future developments need to consider temporal sensor responses. Since fires are dynamic events, authors expected that considering dynamic features would help in the detection of fires, in particular, capturing the dynamic change of oxygen and carbon monoxide [32].

### 6.4. Hierarchical LDA

In another very interesting work performed at Saarland University, researchers developed a system based on a single MOX sensor to reduce false alarms in underground fires, specifically, in coal mines [76]. Although their approach relies on a single MOX sensor, the authors benefit from the fact that MOX sensors exhibit different sensitivity and selectivity when operating at different temperatures, behaving therefore like different virtual sensors. Sensor’s operating temperature can be controlled by applying certain power on a built-in heater placed next to the sensing layer. Briefly, the authors modulated the sensor’s operating temperature and extracted multiple features using a single sensor.

The gas sensor operated in temperature modulation cycle (65-s period function) to increase the sensitivity and selectivity to the target compounds. The temperature profile included temperature ramps and high temperature operation steps. The authors considered several features from the acquired sensor signal. They extracted sensor values at defined temperatures (at discrete times) and slopes of the signal when transitioning between operating temperatures. Extracted features were selected such that, according to previous studies, they are suitable for the discrimination of relevant compounds.

The authors studied thoroughly the scenario of underground fires and identified the volatiles that result from fire (CO and ethane), its ratio (100/1), and the interfering gases (relative humidity, methane, CO, NOX or hydrogen). Based on previous investigations, the researchers designed a measurement profile that simulated, in a laboratory setting, fire and non-fire situations in underground atmosphere. Different concentration levels of CO, C_2_H_4_, NO_2_, H_2_ were presented to the sensor at different humidity (30%, 50%, 70%) background levels. 

Next, they performed a 4-step hierarchical strategy based on Linear Discriminant Analysis (LDA). At each step, the captured data was sequentially classified according to the three levels of humidity (first layer), three levels of methane (second layer), presence of H_2_ or presence of CO or NO_2_ (third classifier), fire/non-fire condition (final classifier). This methodology is equivalent to a decision tree that leads to different final classifiers, the output of which predicts fire, non-fire, or warning situation. The proposed method may be overfitted to the used empirical data since it considers only discrete values of interferences, while in real scenarios, those values will take a continuous distribution.

In a more recent study [2], data acquired in laboratory conditions was compared to field test data. Authors showed that field test data resemble data generated in lab conditions, validating their approach. However, all data was classified as normal operational situation since data only represented “non-fire situations”, i.e., CH_4_, CO and NOx were found at standard concentration levels. The system was operating over several months, which revealed sensor drift. Changing sensor sensitivity in time may make system predictions unreliable as system calibration becomes obsolete. To counteract drift effects, however, authors proposed self-monitoring and self-diagnosis strategies [77]. 

In our view, the above-discussed work provides a very valuable example of using a temperature-modulated sensor to extract various informative features from a single sensor. Using a single sensor, rather than an array of sensors, results in smaller and cost-efficient systems. All in all, the authors performed a very detailed analysis of the scenario and exposed the monitoring system to the relevant volatiles at different humidity levels. The authors developed a 4-step hierarchical classification algorithm that, according to the atmosphere composition, selects the final classifier to predict the presence of fire. This approach seems unpractical when the number of conditions of the environment (the number of interfering gases and concentration levels) increases, for example beyond the restricted scenario of underground mines. The proposed model is not defined when, for instance, the sensors are exposed to 60% RH (which path should the decision model follow? 50% or 70% RH?). In more complex environments, with a larger number of interfering volatiles, it seems more reasonable to build an integral model that considers all the conditions simultaneously and is defined for continuous variables.

## 7. Fire Detectors Exclusively Based on Chemical Sensing

### 7.1. Single Sensor

Already in 1974, Bukowski and Bright, at the National Bureau of Standards (currently known as National Institute of Standards and Technology, NIST, US) explored the feasibility of semiconductor gas sensors to detect fires [78]. They compared a Taguchi gas sensor with photoelectric and ionization fire detectors in small-scale and large-scale setups. They used the same algorithmic approach for smoke detectors and the MOX sensor. Specifically, a signal threshold was defined such that fire condition was signaled when the sensor signal reached the established value. Smoldering fires in the small-scale chamber were carried out for sensitivity comparison at different air flows. Ionization, photoelectric and gas sensor systems triggered the alarm under similar light obscuration conditions. However, when tested on a large room with flaming (shredded paper, wood cribs, gasoline, polystyrene, polyurethane) and smoldering (cotton) fires, gas sensor showed poor sensitivity as it only detected one fire (shredded paper), while ionization and photoelectric detectors detected most of the 26 fires. Authors attributed the inferior performance of MOX sensors to the ventilation of the room that resulted in higher oxygen supply that enabled complete combustion, reducing thereby MOX sensitivity to fire (CO_2_ cannot be detected with MOX sensors).

Obtained results led the authors to draw discouraging conclusions, and they already remarked shortcomings of chemical gas systems, such as long-term drift, and a high number of potential false alarms. Nevertheless, the inferior performance of the chemical system in large-scale setup can be attributed to flame fire tests that induced small quantities of combustion products detectable with MOX sensors. Moreover, more sophisticated data processing algorithms than signal thresholds, adding a variety of gas sensors to the system, and considering other features that capture sensor dynamics could improve the performance of chemical-based detectors.

Some years later, Pfister explored again the feasibility to detect fire with chemical sensors [79]. In particular, he studied the sensitivity of metal oxide gas sensors to the gas concentration levels usually found at early stages of fire. He concluded that combustion products such as CO and hydrocarbons could be sensed for fire detection, although he already pointed at reliability limitations due to cross-sensitivity to humidity.

### 7.2. Neural Networks

Okayama [73] pioneered fire detection using multiple gas sensors and neural networks. Okayama developed two SnO_2_ conductometric gas sensors with different film thickness, and therefore, different sensitivity. The sensors were exposed to volatiles generated from smoldering fires using diverse types of paper, cardboard, cotton, rubber, wood, and polystyrene among others. Volatiles that appear in inhabited environments, such as alcohol-based perfumes, coffee powder, and cigarette butts were also included to test false alarm immunity. The experimental setup was based on a small chamber and a sampling system that brought the volatiles to the sensors.

After confirming the feasibility of fire detection by measuring the sensitivity to the different combustion products, Okayama built a neural network to classify the origin of the detected volatiles. The neural network was composed of two input neurons, five hidden neurons, and two output neurons that represented fire and nuisance probabilities. The instantaneous readings of the two sensors were fed to the input neurons. A definition table with 26 conditions was presented to the neural network. The signals of the same set of experiments were plotted in the sensor space along with model outputs. Such figures enabled sensor signals visualization in a 2-dimension space. Signal trajectories indicated that measurements start in a well-defined area and they spread out in the space according to their nature.

In summary, Okayama confirmed the feasibility to detect combustion products using chemical sensing and therefore detect fire exclusively with gas sensors. Although the pioneering work, he had the vision to explore cross-sensitivities to other volatiles that are present in inhabited environments and he proposed a classifier to discriminate the origin of the detected volatiles. No quantification for fire sensitivity and false alarm immunity could be extracted from the work as the neural network was not evaluated with test experiments.

### 7.3. Hard Rules

Milke et al. studied the chemical composition of combustion products to build models for the discrimination of fire situations and nuisances. Initially, the authors built a 30 × 30 × 150 cm^3^ tunnel in which combustion products or volatiles present in relevant nuisances were introduced through a small aperture. A variety of sensors were integrated into the center of the tunnel: temperature sensor (type K thermocouple), light obscuration sensor (Centronic OSD-100-5T-BNC), CO (Horiba PIR 2000, range 0–1%), CO_2_ (Horiba PIR 2000, range 0–5%) and O2 (Servomex 540A, range 0–20.95%) sensors, and a metal oxide gas sensor (TGS822 Figaro) [69,72].

The authors generated a dataset with 31 experiments that included open flame fires, smoldering fires, heated samples and samples at ambient temperature that were introduced using an atomizer. After signal visualization for each type of measurement, the authors extracted some conclusions: unlike smoldering fires, flaming fires showed CO_2_ concentration peaks higher than 1500 ppm; and smoldering fires exhibited CO concentration levels higher than 28 ppm, which, in turn, was not present in any of the tested nuisances. Based on the extracted conclusions, a set of three rules relying exclusively on chemical sensing was defined to classify the origin of the samples:

**If** CO_2_ > 1500 ppm: **Flaming fire**.
**If** CO > 28 ppm and V_TGS822_ < 6 V: **Smoldering fire**.
**Else**: **Nuisance**.

As it can be noted from the set of rules, temperature and smoke sensors were not used by the classification model. This simple set of rules achieved to correctly classify 28 out of 31 experiments. 

With the same dataset, the authors built a three-layer neural network that incorporated temperature and light obscuration inputs to the considered chemical sensor array (CO, CO_2_ and MOX sensors). The network was composed of six input neurons, six hidden neurons, and three output neurons that indicated flame fire, smoldering fire, or nuisance. After training the network with two-thirds of the data, and testing its prediction with the remaining third, authors improved the classification to 30 out of 31 experiments, being only one smoldering fire misclassified as flaming fire. 

Both classification models, the set of rules and the neural network, relied on CO_2_ concentration for the identification of flaming fires, and non-flaming fires were mainly detected from higher CO concentration levels. However, the authors already expressed some concern regarding the performance of MOX sensors, as they had exhibited lack of response when previously tested in larger setups [78]. This brought the researchers to confirm their promising results at a larger scale setup, although the sensor system was extended to include light obscuration sensors as well (see Section 6).

### 7.4. Fuzzy Logic Rules

In the early nineties, Obayu [80] explored fuzzy logic rules applied to fire detection relying exclusively on chemical sensing. In particular, a multi-sensor system for the detection of catastrophic events in home settings was implemented and integrated with a Z-80 microprocessor. Specifically, the target events included combustible gas leak, carbon monoxide generation, and smoldering fire. The implemented system was composed of a combustible gas sensor (TGS # 109 Figaro), a pair of carbon monoxide gas sensors (# 203, TGS, Japan), and temperature and humidity sensors (HT-150, SOAR). However, the temperature sensor was not considered for the detection of the mentioned events.

The three fire events were simulated in a 44.5-L chamber, while the sensor signals were captured. The combustible gas leak was simulated by introducing liquefied petroleum gas, carbon monoxide was introduced into the chamber to reach a concentration of 180 ppm to simulate carbon monoxide generation, and smoldering fire was simulated by setting fire on a piece of a cotton cloth. Moreover, cigarette smoke was also introduced in the measurement chamber to include nuisance scenario in the dataset.

Based on the observation of the signals when the sensors were exposed to the different target scenarios, a fuzzy set of rules was built to identify the type of event:

- *IF* Combustible Gas Sensor is **very high** *AND* Carbon Monoxide Gas Sensors are **high** *AND* Humidity Sensor is **slightly high**, *THEN* Smoldering Fire occurs.
- *IF* Combustible Gas Sensor is **very high** *AND* Carbon Monoxide Gas Sensor is **rather high**, *THEN* Combustible gas leak occurs
- *IF* Combustible Gas Sensor is **high** *AND* Carbon Monoxide Gas Sensors are **very high**, *THEN* Carbon Monoxide Generation occurs.

The model showed some limitations to identify the type of event, in particular, it showed poor ability in differentiating between smoldering fire and smoke from cigarettes, which would yield to a large number of false alarms. Only one repetition of each event was considered to build the set of fuzzy rules. Therefore, the repetitivity of the sensor responses could not be evaluated.

Moreover, the rules were built after the system was placed in the measurement chamber and the sensor responses acquired. To what extent the intensity of the sensor responses (low, normal, high, etc.) depends on the volume of the chamber or the induced concentration levels in the chamber remained unexplored. This is particularly needed as rooms in home settings are orders of magnitude larger than the employed test chamber, and a wide range of concentration levels can be induced by gas leaks, fires or carbon monoxide sources. Müller and Fisher proposed the use of fuzzy logic to process signals acquired with smoke and temperature sensors. They stated the need for large datasets to properly set the fuzzy rules. They simulated 8 years of data, which allowed the optimization of their model [81]. This points out the difficulties of extending a set of fuzzy rules to account for different environments, fire types and backgrounds.

Nevertheless, it is remarkable the fact that in this work, Obayu went beyond a mere study on sensor sensitivity and proposed a classification algorithm for fire identification.

In a very recent work, Mobin et al. proposed an intelligent system for fire detection that combines a multi-sensor system and fuzzy logic rules [82]. The system includes two flame sensors, two gas sensors and one temperature and humidity sensor. The sensor data were acquired using an Arduino UNO (Italy). Basically, when the signals of the sensors are high, the algorithm processes the sensor data and decides if there is a fire situation. If a fire situation is in progress, the system activates the control circuit of the extinguisher and mitigates the fire. The experiments were done using a cigarette lighter to emulate a fire situation. The algorithm is capable to detect 95% of the flames presented to the system. Even when the work is useful to study the feasibility of intelligent systems to detect and mitigate fire, further work is required to explore the reliability and robustness of the system in more complex fire scenarios.

### 7.5. Principal Component Analysis and Nearest Neighbours Classifiers

Ni et al. proposed a methodology based on a k-nearest neighbor after dimensionality reduction [83]. They focused the interest on the scenario of electrical fires, which is particularly favorable for gas-based fire detectors. High intensity flowing through electrical cables may be a sign of early fire condition. However, high-temperature excursion may be required until insulation materials (typically thermal resistant materials) release quantities of smoke that smoke detectors can detect. On the other hand, during pre-combustion, vapor generation happens before smoke formation, and therefore, gas sensors can detect released volatiles before a significant amount of smoke is produced. As a result, chemical-based detection systems are especially well suited to provide early detection of electrical fires.

In their work, Ni et al. tested several materials that are used as wire insulation (PVC, Teflon^®^, Kapton^®^, and silicone rubber). Electrical failure was simulated by inducing thermal excursions on the materials. 15-cm length pieces of wire were used for each measurement, and the minimum power (between 6.1 W and 13 W) that released volatiles was applied to each sample type. The baseline was acquired for 3 min, after which thermal excursion was carried out for 5 min. Four replicates of each sample type were heated up, and the released volatiles were presented to the sensors, for a total of 16 measurements.

Different sensing technologies were studied, including electrochemical sensors, quartz microbalance sensors with different polymer coatings, and metal oxide sensors. After sensitivity tests, eight MOX and three electrochemical sensors were selected to build the classification model. Specifically, sensor signals at specified time points were selected to build the classification model. Authors used acquired baseline at the beginning of each experiment to compensate baseline shifts. After feature normalization, a classification algorithm was performed based on Principal Component Analysis coupled to K-nearest neighbor. PCA reduced the dimension of the data to two dimensions, and K-NN was used to predict the type of sample (wire insulation) under thermal stress. The performance of the model was evaluated with leave-one-out methodology. Although the simplicity of the classification methodology, the authors achieved 100% classification accuracy. It is important to note, although, that if the normalization step was omitted, only 82% of the samples were classified correctly.

The authors proposed a methodology to classify wires by the insulation type. This scenario does not correspond exactly to fire detection, as other important considerations such as false alarm immunity and change of environmental conditions were not included in the analysis. However, they provided an example of the importance of feature selection and data pre-processing, as feature normalization was necessary to obtain higher classification accuracy. Nevertheless, electrical fire is definitely a scenario in which chemical-based systems can show their superior performance with respect to smoke detectors and, therefore, it needs further research, including the study of false alarm immunity and other fire conditions.

### 7.6. Other Approaches

Sawada et al. studied fire detection using exclusively MOX gas sensors [84]. Specifically, they placed 8 MOX gas sensors of the same type (TGS#800, Figaro) in a 55 m^3^ test room. The eight sensors were distributed in the measuring room at different distances from a source of volatiles. Four scenarios were tested in the room, with three repetitions each: person smoking, cigarette smoke, burning cigarette end on a cotton cloth, and burning cigarette end on a curtain.

The authors explored the feasibility to group the data by measurement type. After sensors’ signals were filtered to reduce noise, two features were considered using only the two sensors closer to the source. The first feature was the sensor reading (amplitude of the signal) one minute after the measurement started. The second feature was the slope of the linear fit between the signals acquired with the two sensors. Using the repetitions for each case, they built scatter plots: the amplitude of the signal versus the slope of the fitted function. They found that data points that correspond to a person smoking appeared in a different region than the rest of the scenarios.

**Figure 10:**
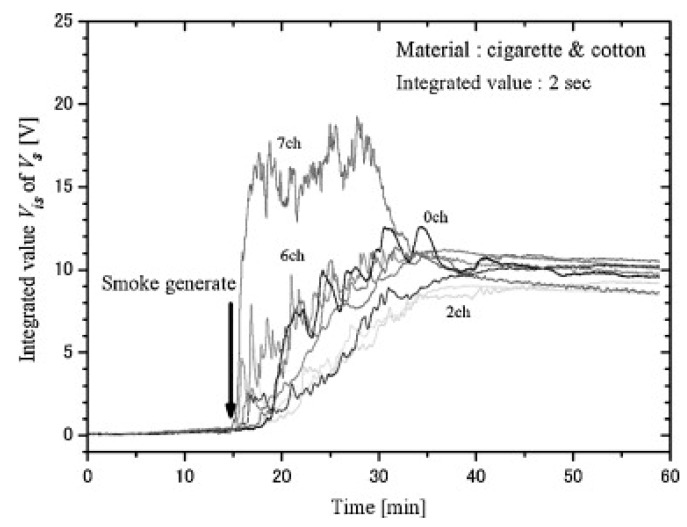
Integrated sensor signals (in 2-s windows) under smoldering fire condition. The dynamics of the gas plume are different for sensors located at different distances of the source. Shifted-temporal signal correlations between sensors placed at different locations are expected and they can be used to improve the prediction ability of the classification model. 7ch is the sensor closer to the source; 0ch is the sensor further from the source. The delay that corresponds to the time needed for the volatiles to reach sensors located further from the source is observed in the figure. Reproduced from [84].

Authors did not build a classifier for the detection of fire or identification of fire types. However, several conclusions can be drawn from their work. Gas plume dynamics may help to differentiate fire from nuisances. Interestingly, the authors found different dynamics of the sensor signals at various locations: sensors placed close to the fire source showed faster fluctuations as they are more sensitive to gas plume movements. As volatiles tend to travel in patches, shifted-temporal signal correlations between sensors placed at various locations may be expected. These correlations may help to corroborate (or distrust) sensor predictions and thereby improve false alarm immunity (see Figure 10).

The authors also did not implement any temporal correction on the signals captured from the differently located sensors. However, in the presented figure, one can observe a delay in the response of the sensor placed further from the source. This delay can be used as well to provide additional information on the position of the fire source.

In a recent work, Krüger et al. presented a MEIS hydrogen sensor for fire detection applications and performed several fire experiments [85]. The experiments were performed in two different scenarios; in a smoke chamber inspired by the ISO 5659-2 and in a 2-room apartment with similar proportions than the ratios specified in the EN 54. The experiments performed in the chamber correspond to polymeric materials: Polyethylene, polyurethane and wood. To test the sensors under real-working conditions, they burned different materials in the apartment such as carpet, kitchen roll, kitchen sponge, cheese and armchair. In both scenarios, they observed that H_2_ was released in the early stages of the fire experiments (before smoke). Also, the sensor responses were different depending on the materials and scenarios.

Recently, Adib et al. [86] presented an interesting work for fire detection using a chip that integrates 16 SnO_2_ nanowires gas sensors and classification algorithms based on Linear Discriminant Analysis (LDA). To test their system, they performed tests of smoldering PBC, Bench and cotton using a hot plate. The experiments were performed in a chamber and in a big container. They obtained a classification rate of 88% in experiments performed in the chamber and 86% in the experiments performed in the container. Further work is required to explore the robustness and the generalization of the model using an extensive dataset.

Lee et al. developed NiO, SnO_2_, WO_3_ and In_2_O_3_ NCs nanocolumns gas sensors using the glacing angle deposition technique (GLAF) [87]. The sensors were designed for fire detection. To study the behavior of their sensors under fire conditions, they performed smoldering PVC fire experiment. Their methodology consists of heating 5 g of PVC plastic and increasing the temperature from 5 °C to 350 °C. They observed that different gases were emitted depending on the temperature of the hot-plate. Additionally, the response time of sensors was much faster than the smoke sensor. They concluded that their developed sensors are able to detect PVC fire and identify different stages of PVC combustions.

Finally, Courbat et al. developed a colorimetric CO sensor based on a rhodium complex [88]. The sensor relied on the chemochromic properties of the reagent when exposed to CO: its color changed from purple to yellow when CO concentration level increases. The colorimetric film was integrated with LED and photodetectors. The sensitivity of the device was explored with test fires inspired by EN-54 standard (TF2, TF3, TF5) downscaled in a 1 m^3^ volume chamber. The system showed sensitivity to the tested scenarios, and sensor showed baseline recover after some minutes. However, cross-sensitivity to other volatiles and scenarios was not evaluated, and, therefore, the viability of the device remained uncertain.

## 8. Summary and Conclusions

The use of gas sensors for fire detection has both strengths and weaknesses. The possibility to detect toxic emissions from fires before actual smoke reaches the detector is a remarkable strength with respect to conventional fire detectors. Earlier warning to building occupants may lead to better protection against intoxication, incapacity and, ultimately, death. This path has been already explored with the integration of carbon monoxide electrochemical cells in multisensor systems for fire detection. However, the range of toxicant emissions from fire, plastic overheating or new building materials covers many other volatiles beyond carbon monoxide. Consequently, the inclusion of chemical sensor arrays to detect other hazardous compounds deems necessary. While this is possible, and it can lead to higher fire sensitivity and earlier fire detection, it can come at the expense of less reliable predictions. Actually, high rate of false alarms constitutes a downside for gas-based fire detectors. This is a direct consequence of the poor selectivity of low-cost solid-state sensors, which are also sensitive to volatiles generated during normal daily activities, such as cleaning or cooking, for example. For this reason, the only path to improve false alarm immunity is the use of pattern recognition algorithms that could differentiate between sensor signatures induced from fire or nuisance scenarios. While this can be accomplished by a large variety of soft-computing and machine learning methods, it requires extensive and time-consuming testing since fire conditions and nuisance scenarios can be extremely diverse. The reviewed literature shows that the number and type of nuisances proposed by authors are also large. Standardization specifically tailored for fire detectors based on chemical sensors lags, and efforts are required to find a consistent set of testing conditions that ensures the robustness of detectors against nuisances. Additional problems may appear due to sensors drift or sensor to sensor tolerances, but this was beyond the scope of this review work. 

## Figures and Tables

**Table 1:** 30 min LC_50_ values for rats [5].

Product	30 min LC_50_ in ppm
CO	5700
HCN	165
HCl	3800
HBr	3800
HF	2900
SO_2_	1400
NO_2_	170
Acrolein	150
Formaldehyde	750

**Table 2:** Measured CO concentration and CO dose (accumulated since test start) at shortest time of alarm (top) and at mean time of alarm (bottom). Values in **bold** are above the ID_50_ limit. The photoelectric smoke detector did not trigger the alarm for three of the performed fire measurements. CO concentration is consistently higher at the time of activated alarm for photoelectric smoke detector, reaching values above the ID_50_ limit. Data extracted from SP Fire Research Experiment Norway [42].

	Photoelectric		Detector with CO Sensor	
	CO Concentration (ppm)	CO Dose (ppm minute)	CO Concentration (ppm)	CO Dose (ppm minute)
Shortest time of alarm	576	30,859	25	587
	733	31,384	38	425
	502	17,855	53	1121
	639	22,690	52	802
	643	18,985	35	276
	993	**57,893**	36	515
	-	-	44	965
	-	-	30	437
	-	-	47	902
Mean time of alarm	664	**37,593**	35	875
	1453	**63,957**	42	766
	638	24,371	62	1236
	907	**39,325**	61	965
	933	32,547	35	315
	1075	**64,184**	37	554
	-	-	46	1019
	-	-	36	489
	-	-	46	960

**Table 3:** Standard Test Fires described in the EN-54 standard.

Fire	Type
TF1	Open wood fire
TF2	Rapid *smoldering pyrolysis* wood
TF2a	Slow smoldering pyrolysis wood
TF2b	Smoldering pyrolysis wood
TF3	Rapid *smoldering* cotton
TF3a	Glowing slow *smoldering* cotton
TF3b	Glowing *smoldering* cotton
TF4	Open plastics fire (Polyurethane)
TF5	Liquid fire (n-heptane)
TF5a	Small n-heptane fire
TF5b	Medium liquid n-heptane fire
TF6	Liquid fire (ethyl alcohol)
TF7	Slow *smoldering* wood
TF8	Low temp. liquid fire (decalin)
TF9	Deep-seated *smoldering* cotton

**Table 4:** Concentration measurement ranges (in ppm) for fire emissions provided by different vendors.

Gas	IST	Alphasense	GfG
NH_3_	√ 10 ppm	√ 100 ppm	√ 200 ppm
CO	√ 300 ppm	√ 500 ppm	√ 300 ppm
H_2_	√ 2000 ppm	√ 2000 ppm	√ 2000 ppm
HCl	√ 30 ppm	√ 100 ppm	√ 30 ppm
HCN	√ 30 ppm	√ 100 ppm	√ 50 ppm
HF	√ 10 ppm		√ 10 ppm
HBr			√ 30 ppm
H_2_S	√ 30 ppm	√ 100 ppm	√ 100 ppm
NO	√ 100 ppm	√ 100 ppm	√ 100 ppm
NO_2_	√ 50 ppm	√ 20 ppm	√ 30 ppm
SO_2_	√ 100 ppm	√ 20 ppm	√ 10 ppm
O_2_			25%

**Table 5:** Availability of electrochemical cells for the detection of toxics ^1^.

Gas	Honeywell	Casella	Draeger	Geotech	IS	Ion Science	MSA
NH_3_	√	√	√	√	√		√
CO	√	√	√	√	√	√	√
H_2_	√	√	√	√	√		
HCl	√	√	√	√	√		√
HCN	√	√	√	√	√		√
HF	√	√					√
HBr	√	√					√
H_2_S	√	√	√	√	√	√	√
NO	√		√				
NO_2_	√	√	√	√	√		
SO_2_	√	√	√	√	√		√
O_2_	√	√	√	√	√	√	√

^1^ IST: International Sensor Technology (http://www.intlsensor.com/); GfG: Innovative Gas Detection Technology (http://www.gfg-inc.com/); Alphasense: (http://www.alphasense.com/); Honeywell: (http://www.honeywellanalytics.com); Casella: (http://www.casellasolutions.com); Draeger. (http://www.draeger.com); Geotech (http://www.geotechuk.com); IS: Industrial Scientific (http://www.indsci.com); MSA: (http://www.MSAsafety.com).

**Table 6:** Confusion matrices for the multisensor system with 2 MOX, CO, CO_2_, T and light obscuration sensors with dimensionality reduction and hard rules (top, from [63]); the multisensory system with 2 MOX, CO, CO_2_ and T with hard rules (middle, from [62]); the commercial smoke detector (bottom, from [62]).

**MOX (x2), CO, CO_2_, Light, T + PCA and Hard Rules**	**Flaming Fire**	**Smoldering Fire**	**Nuisance**
Flaming fire	34	-	-
Smoldering fire	-	14	2
Nuisance	-	10	27
**MOX (x2), CO, CO_2_, T + Hard Rules**	**Flaming Fire**	**Smoldering Fire**	**Nuisance**
Flaming fire	34	-	-
Smoldering fire	-	10	6
Nuisance	-	5	32
**Smoke Detector + Threshold**	**Fire**	**Non-Fire**	
Flaming fire	26	8	
Smoldering fire	8	8	
Nuisance	4	33	

**Table 7:** Rose-Pehrsson et al. considered a very complete set of fire/nuisances scenarios, with various repetitions of each, for a total number of 240 measurements (120 background, 82 fires and 38 nuisance sources). Table adapted from [74].

Fire/Nuisance	Id	Description
F	1	Propane burner
F	2	Heptane pool fire
F	3	JP-5 pool fire
F	4	JP-8 pool fire
F	5	Alcohol pool fire
F	6	Smoldering mattress
F	7	Flaming mattress foam only
F	8	Flaming mattress loose bedding
F	9	Flaming mattress tucked bedding
F	10	Smoldering pillow
F	11	Smoldering electrical cable, LSDSGU-14: cross-linked polyolefin jacket, silicon rubber insulation
F	12	Smoldering electrical cable, LSTHOF-9: cross-linked polyolefin jacket, ethylene propylene rubber insulation
F	13	Smoldering electrical cable, LSTPNW-1 1r2: cross-linked polyolefin jacket, cross-linked polyethylene insulation
F	14	Igniting electrical cable, LSDSGU-14: cross-linked polyolefin jacket, silicon rubber insulation
F	15	Igniting electrical cable, LSTHOF-9: cross-linked polyolefin jacket, ethylene propylene rubber insulation
F	16	Igniting electrical cable, LSDSGU-50: cross-linked polyolefin jacket, silicon glass insulation
F	17	Office trash can fire
F	18	Pipe insulation NH Armaflex exposed to a propane fire
F	19	Pipe insulation coated with oil NH Armaflex exposed to a propane fire
F	20	Pipe insulation calcium silicate exposed to a propane fire
F	21	Pipe insulation coated with oil calcium silicate exposed to a propane fire
F	22	Polyimide acoustic insulation exposed to a propane fire
F	23	Nomex honeycomb wall panel TODCO exposed to a propane fire
F	24	Nomex honeycomb wall panel Hexcel exposed to a propane fire
N	1	Burning toast
N	2	Normal toasting
N	3	Welding
N	4	Cutting steel with acetylene torch
N	5	Grinding steel
N	6	Grinding cinder block
N	7	Cutting loan board wood
N	8	Burning popcorn in microwave
N	9	Gasoline engine exhaust
N	10	Electric heater and halogen lamps
N	11	People talking and moving around in the test compartment
N	12	Cigarette smokers
